# DisPredict: A Predictor of Disordered Protein Using Optimized RBF Kernel

**DOI:** 10.1371/journal.pone.0141551

**Published:** 2015-10-30

**Authors:** Sumaiya Iqbal, Md Tamjidul Hoque

**Affiliations:** Department of Computer Science, University of New Orleans, New Orleans, LA, United States of America; UMR-S665, INSERM, Université Paris Diderot, INTS, FRANCE

## Abstract

Intrinsically disordered proteins or, regions perform important biological functions through their dynamic conformations during binding. Thus accurate identification of these disordered regions have significant implications in proper annotation of function, induced fold prediction and drug design to combat critical diseases. We introduce DisPredict, a disorder predictor that employs a single support vector machine with RBF kernel and novel features for reliable characterization of protein structure. DisPredict yields effective performance. In addition to 10-fold cross validation, training and testing of DisPredict was conducted with independent test datasets. The results were consistent with both the training and test error minimal. The use of multiple data sources, makes the predictor generic. The datasets used in developing the model include disordered regions of various length which are categorized as short and long having different compositions, different types of disorder, ranging from fully to partially disordered regions as well as completely ordered regions. Through comparison with other state of the art approaches and case studies, DisPredict is found to be a useful tool with competitive performance. DisPredict is available at https://github.com/tamjidul/DisPredict_v1.0.

## 1 Introduction

Many protein regions and some entire proteins do not adopt well-defined, stable three-dimensional (3D) structures in an isolated state and under different non native environments [1–3]. These proteins or partial regions of proteins are called intrinsically disordered proteins (IDPs) or disordered regions in proteins (IDRs), also known as natively unstructured, denatured or unfolded. The coordinates of their backbone atoms have no specific equilibrium states and can vary largely due to variable physiological conditions, and thus adopt dynamic structural ensembles. Structurally, IDPs (or IDRs) encompass proteins or protein-regions with extended disorder, collapsed disorder and semi-collapsed disorder. These reflect differences in the underlying biophysical characteristics including low hydrophobicity and high net charge, marginal level of residual secondary structure [4, 5], dynamic side chains and secondary structures [6, 7], rapidly exchanging backbone side-chain hydrogen bonds which make a region unable to form specific secondary structure [3]. Recognition of these protein disordered regions is important for appropriate protein structure prediction, disease causing protein identification, proper annotation of function, induced folding and binding region prediction.

For the last two decades, many works have been presented in evidence that many proteins do not follow the well-known paradigm of sequence to stable structure to function. Rather these proteins adopt disordered state for complex and essential biological functions [1, 6, 8, 9] such as cell cycle control and cellular signal transduction, transcriptional and translational regulation, membrane fusion and control pathways [1, 10, 11]. They participate in molecular recognition, molecular assembly and protein modification [12, 13] via protein-protein, protein-nucleic acid and protein-ligand interactions as well. Disorder proteins are found to be highly associated with critical human diseases [14–16], such as cancer, amyloidoses, cardiovascular and neurodegenerative diseases, genetic diseases. Thus, identifying them assists in effective drug development [17, 18].

In reality the IDPs are abundant. Approximately 70% of the structures released by Protein Data Bank (PDB) [19] contain some disordered residues [20, 21]. A curated database of disordered proteins, called DisProt [22] contains annotation for 694 protein sequences and 1539 disordered regions in its current version 6.02. The IDEAL [23, 24] and MobiDB [25, 26] databases also provide useful collections for annotation of intrinsic disorder. PDB [19] database, which gives provision of finding disordered regions in the solved secondary or tertiary structure incorporates 105,097 protein entries. To compare, the overall number of non-redundant protein sequences is 46,968,574 according to the most recent 68 release of RefSeq database [27]. However, due to highly flexible characteristics of the residues of IDRs or, IDPs [28]), experimentally verified annotation of intrinsic disorder is growing slowly. Thus to keep pace with this large-scale increase in protein database, effective computational methods for correct identification of disordered residues in IDPs or, IDRs are necessary.

Several computational methods have been developed to fulfill the fast annotation requirements for the rapidly growing known protein sequences. Machine learning based some of these well-known approaches are PONDR series [20, 29, 30], DISOPRED [31], DISOPRED2 [32], DisEMBL [33], DISpro [34], RONN [35], Spritz [36], PROFbval [28, 37], DisPSSMP [38, 39], PrDOS [40], POODLE series [41, 42], NORSnet [43], IUP [44], OnD-CRFs [45], PreDisOrder [46], SPINE-D [47] and ESpritz [48]. Several existing tools, for instance GlobPlot [49], IUPred [50], FoldIndex [51] and Ucon [52], usage knowledge such as the relative composition and propensity of amino acids. On the other hand, DISOclust [53] is based on the analysis of how disorder is related with protein folding and uses predicted three-dimensional structural characteristics. Combination of individual methods in a complementary method gave raise to effective disorder predictors, such as metaPrDOS [54], MD [55], MFDp [56], PONRD-FIT [21] and very recent MFDp2 [57].

In this article, we propose a new disorder predictor, named “DisPredict (Disorder Predictor)” [58]. DisPredict classifies ordered and disordered residues in a protein sequence with higher accuracy, specifically in terms of Mathews Correlation Coefficient (MCC) and Area Under the receiver operating characteristics Curve (AUC). Dispredict is based on Support Vector Machine (SVM) using Radial Basis Function (RBF) as kernel. We further strengthened the classification performance of DisPredict by selecting optimized parameters of SVM which significantly improved the performance. We utilized a comprehensive set of 56 features to characterize disorder in protein sequence. We compared DisPredict’s performance with existing predictors, SPINE-D [47] and MFDp [56], followed by an analysis of its performance with respect to different types of amino acid, length of disorder region and datasets.

## 2 Materials and Methods

In this section, we discuss the data-sources, data-processing, input-feature generations, software design and platform and performance evaluation.

### 2.1 Preliminary Disordered Data Sources

In the prior studies, PDB [19] and DisProt [22] are considered as the primary repositories of IDPs. Disorder regions are composed of residues with missing coordinates in structure solved by X-ray crystallography, whereas the residues show highly variable coordinates within ensemble solved by NMR. We selected two datasets which combine sequences from PDB having disordered residues without coordinates (recorded in REMARK 465) and sequences from DisProt, having curated annotations of disorder regions including properties such as short (≤ 30 residues) and long (> 30 residues) disordered regions, partial as well as fully ordered or disordered chains.

### 2.2 Datasets

We used two different datasets, MxD and SL, to train, test and cross-validate our proposed DisPredict. MxD and SL datasets were used to train two disorder predictors, SPINE-D [47] and MFDp [56], respectively. We collected and utilized these datasets to be able to consistently compare DisPredict with these two predictors.

The Mixed Disorder (MxD) dataset is a combination of protein sequences with disordered residues from both PDB and DisProt. Originally developed MxD dataset [56] has 514 protein sequences including 205 chains from PDB and 309 chains from DisProt. We carried out further purification by removing sequences with unknown amino acid (X-tag) since they do not have specific physicochemical properties to get corresponding features in our methodology. This led to the MxD444 dataset, with 444 chains and 214,054 residues, that mixes 49,090 (about 23%) disordered residues and 164,964 (about 77%) ordered residues.

SL477 dataset was prepared by the developers of SPINE-D predictor from the benchmark SL (Short Long) dataset [59]. The SL dataset encompasses short and long disordered regions as well as ordered regions. It was built by re-annotating the sequences extracted from DisProt to include reliable order and disorder contents. Among the annotated regions in the SL dataset, 50% of the regions are of the short-disordered category. The short regions in SL dataset are of length 20 residues or less [59]. It is important to incorporate this disorder annotation in a dataset since these short disordered regions are found functionally important as they obtain induced folding with the close proximity of appropriate partners. SL477 also includes very long disordered regions as well as completely disordered proteins, called intrinsically disordered proteins (IDPs). SL dataset comprises of proteins with disorder regions annotated by NMR experimental method as well. To achieve combination of sequences with low sequence identity, SL dataset’s sequences were clustered and filtered using BLASTCLUST [60] which resulted in 477 chains with < 25% sequence identity between each pair. SL477 has total 215,343 residues, of which 56,887 (about 25%), 72,808 (about 34%) and 85,648 (about 40%) residues are annotated as disorder, order and unknown, respectively. Unknown residues are those which are marked unknown in the source datasets. We disregarded the residues with unknown annotation during both in training and in evaluating our proposed approach.

Moreover, to test our predictor with less overlapped sequences from training dataset, we extracted two independent test datasets from the two training datasets using BLASTCLUST [60]. We filtered 171 protein chains from SL477 datasets with less than 10% similarity with any sequence from MxD444 dataset. We call these 171 protein chains with 42,572 residues as SL171. We used SL171 as test dataset to independently test our predictor’s performance while it is trained by MxD444 dataset. Similarly, we extracted 134 sequences from MxD444 dataset that are independent from SL477 dataset at 10% identity cut off. We call these 134 protein sequences with 38,823 residues as MxD134. We utilized MxD134 as test dataset to independently test our predictor’s performance while it is trained by SL477 dataset.

Further, we prepared a completely new dataset that is completely independent of the training sets of DisPredict, SPINE-D [47] and MFDp [56]. We collected 48 new protein chains from DisProt [22] released after version 5.1 upto current version of 6.02. These protein sequences were combined with another 25 protein chains culled from PDB [19]. Protein chains were extracted from PDB x-ray structures with resolution ≤ 3.0 angstroms, length ≥ 50, sequence identity cut-off of 30% and by choosing single chain proteins. We randomly selected 25 chains from the output of this experiment so that no sequence is more than 25% similar with the training sequences. To have a proper combination of ordered and disordered proteins, we ensured that none of these 25 proteins can contain disordered residues expect terminal regions. Altogether, it gave us 73 protein sequences which is a combination of 37 full disorder chains, 23 full ordered chains and 13 protein chains with disordered and ordered regions. We call this Disorder Dataset as DD73. DD73 dataset allows us to perform a robust comparison among DisPredict, SPINE-D [47] and MFDp [56], as it is independent of both SL and MxD dataset.

### 2.3 Input Features

Our input features were carefully chosen to be able to include useful properties such as the sequence information, evolutionary information as well as the structural information (listed in Table 1). Studies suggest that necessary information for the correct folding of a protein is encoded in its amino acid sequence including disorder contents [31]. Moreover, disordered regions are abundant in low complexity regions and in regions with low content of hydrophobic amino acids [28, 61]. The physicochemical properties [62] of amino acid are also found to have some degree of correlation with the length of disordered regions; as short disordered regions are mainly negatively charged while long disordered regions are nearly neutral [28, 55]. These observations motivated us to use amino acid type (AA), indicated by one numerical value out of twenty and seven physicochemical properties (PP) as features to predict disordered residues in our proposed approach.

**Table 1:** List of features used in DisPredict.

Feature Category	Feature Count
Amino Acid (AA)	1
Physicochemical Property (PP)	7
PSSM Profile (PSSM)	20
Secondary Structure Content (SS)	3
Accessible Surface Area (ASA)	1
Torsion Angle Fluctuation (Φ, Ψ)	2
Monogram (MG)	1
Bigram (BG)	20
Terminal Indicator (T)	1
Total	56

Disordered regions and their related functions are conserved within the sequence during evolution [63], thus we considered position specific scoring matrix (PSSM) as input features to capture evolutionary information. PSSM (sequence length × 20) was generated for each sequence by executing three iterations of PSI-BLAST [60] against NCBI’s non-redundant database [27, 64]. The PSSM values were normalized further using numeric value nine [65], which we call as *PSSM normalizing factor*. We employed sequence based predicted secondary structure (SS) probabilities for helix, sheet and coil residues [65], predicted solvent accessibility (ASA) [66] and predicted backbone dihedral torsion angles (Φ and Ψ) fluctuations [67] as features. We included these six features since disordered residues can be characterized by the lack of stable secondary structure [7, 8, 28] and also the unstructured regions are found to have large solvent accessible area [68].

**Fig 1:**
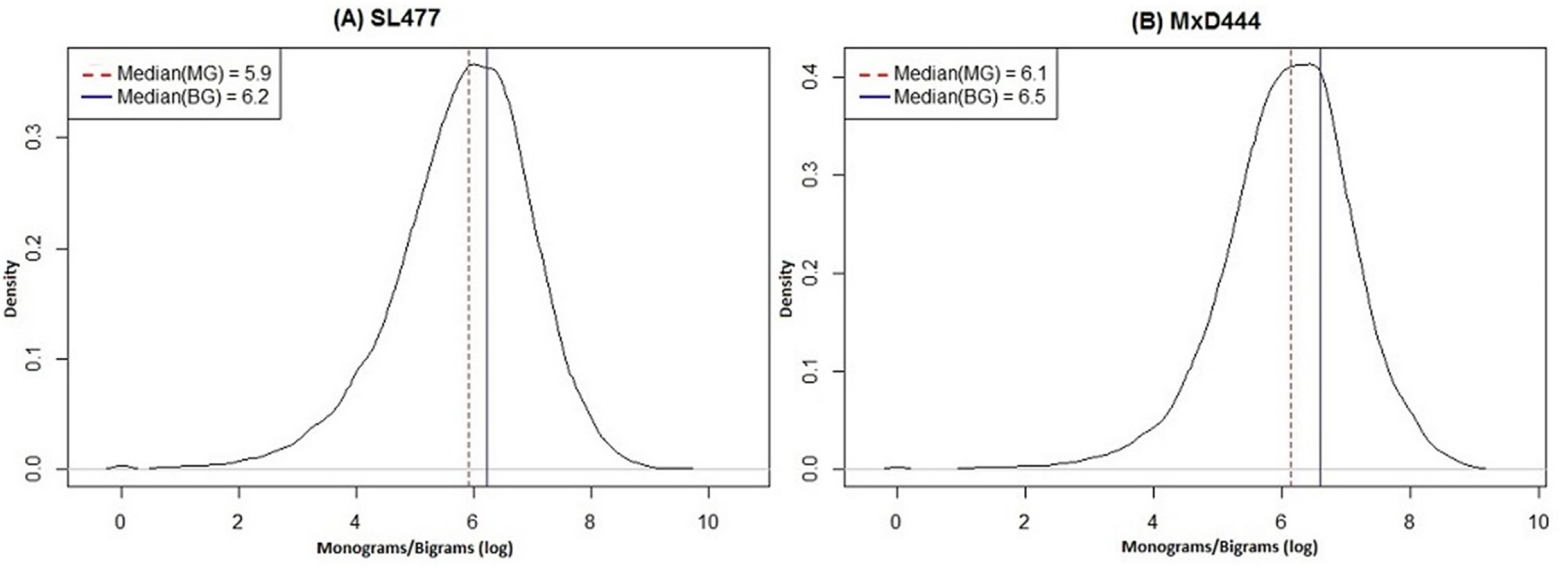
Density distribution curves of monograms and bigrams for (A) SL477 and (B) MxD444 dataset. The x-axis and y-axis show the monograms/bigrams in logarithmic scale and density index of the distribution, respectively. For each figure, the dotted (*red*) and solid (*blue*) vertical lines correspond to median values of the distribution for monograms (MG) and bigrams (BG), respectively.

Literature suggests that the conserved evolutionary information given by PSSM can be transformed from primary structure (amino acid sequence) level to three dimensional structure level by computing monograms and bigrams from PSSM values [69]. The monogram-bigram probabilities characterize the subsequence of a protein sequence that can be conserved within a fold in terms of transition probabilities from one amino acid to another [70]. Thus the monogram-bigram features are useful in identifying the evolutionary folded (ordered) or, unfolded (disordered) region of proteins, which motivated us to utilize them as features in disorder prediction. We computed monogram feature matrix (1 × 20) and bigram feature matrix (20 × 20) for each sequence from its PSSM. Monogram feature matrix consists of one monogram value (MG) for each type of amino acid and bigram feature matrix consists of one bigram value (BG) for each pair of 20 possible amino acids, respectively. Further, our analysis based on multiple datasets collected from PDB and DisProt shows that both the monograms and bigrams follow a normal density distribution in their logarithmic space with approximately consistent median value equals to 6.0 within any dataset (Fig 1). Therefore, we used exp(6.0) to normalize these values and reduce the noise. To distinguish the terminal residues for their position specific disorder like behavior, we included terminal indicator feature (T) by encoding five residues of N-terminal as {−1.0, −0.8, −0.6, −0.4, −0.2} and C-terminal as {+1.0, +0.8, +0.6, +0.4, +0.2} respectively, whereas rest of the residues were labeled 0.0. Note that, we included the fundamental features to characterize disorder in protein in our feature set which are well studied and utilized in the literature [47]. Further, we enhanced the feature set by including new features, like MGs and BGs.

We included the information of neighboring residues within the features of each residue by using a sliding window, keeping the target residue at the center of the window. The motivation was to incorporate the native interactions and contacts of neighboring residues which are found to play essential roles in determining protein structures and protein folding dynamics [71, 72]. We determined the 10-fold cross-validation performance of DisPredict for 13 different window sizes (1, 3, 5, …, 23, 25) to find the optimal window size 21. Thus, there were 1176 (since, *window* *size* × *total* *feature* *count* = (21 × 56) = 1176) features used for each residue. The features were finally scaled within the range [−1, + 1] before using.

### 2.4 SVM Design and Parameterization

**Fig 2:**
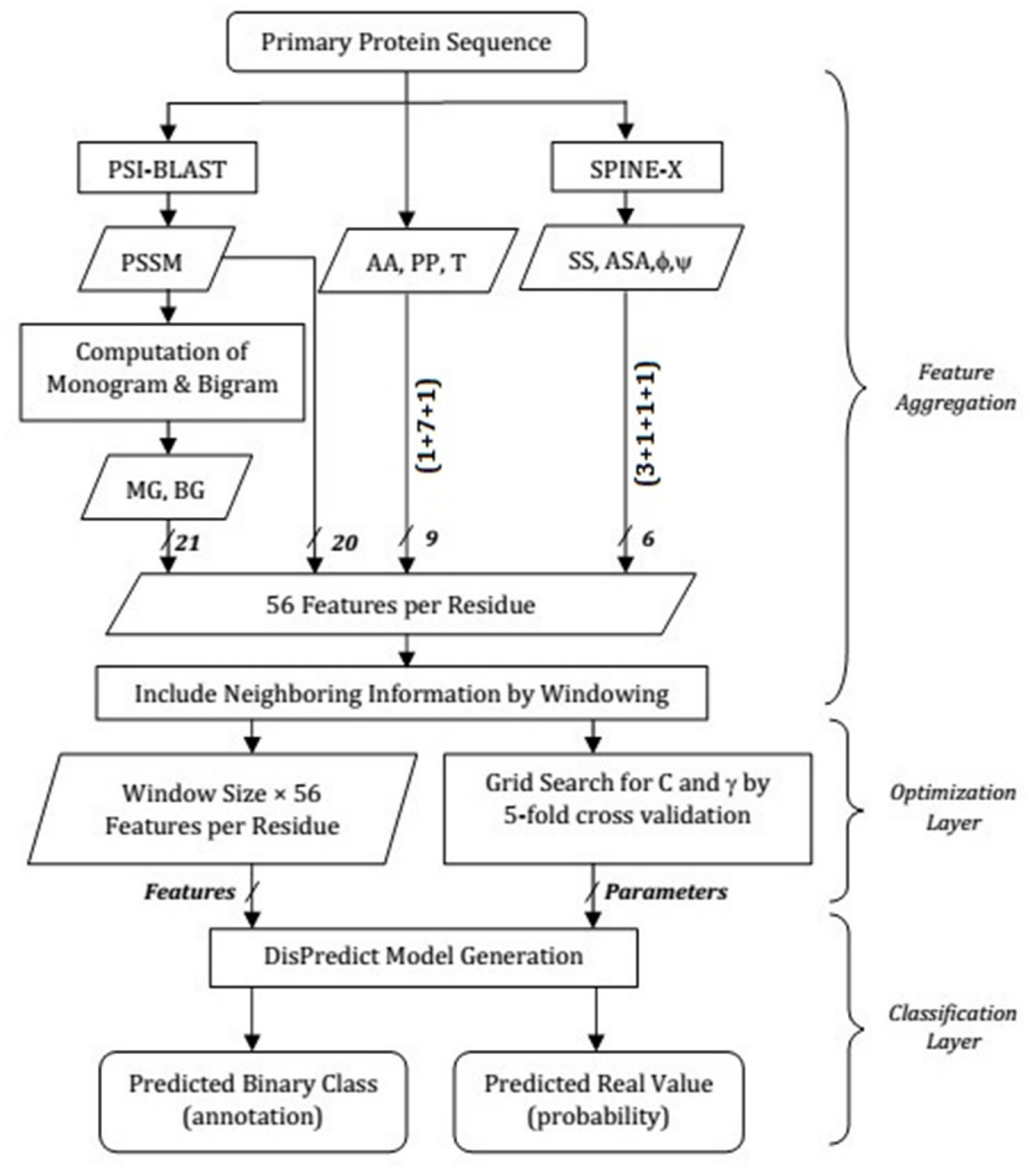
Overview of feature aggregation, optimization-layer and classification-layer in DisPredict. In the feature aggregation step, features are shown in their abbreviated form according to Table 1 and the arrows are labeled by the number of features involved. The classification-layer receives final feature set from the feature aggregation step and optimal parameters from the optimization-layer. Then, it generates the predictor model and outputs both binary annotation and real-valued class probabilities.

DisPredict is a two-layer disorder predictor that integrates *optimization-layer* and *classification-layer*. The classification-layer is developed using a single support vector machine (SVM), namely LIBSVM [73]. Due to the working principle of SVM of simultaneously minimizing the empirical classification error (training error) and generalized error (test error) by maximizing the geometric margin of the separating hyperplane, it can be regarded as an effective technique in hard classification problems specially in bioinformatics and computational biology area. We used Gaussian or, radial basis function (RBF) kernel for the SVM to extend its capability to handle non-linearly separable classes. RBF transforms the input feature space into infinite dimension space (*i.e.* Hilbert space), which results in a linear separating hyperplane. On the other hand, in the optimization-layer of DisPredict, we selected two parameters, *C* and *γ*, where *C* is the cost of misclassification and *γ* is the parameter of fitting best mode of RBF. The optimal values for the parameters *C* and *γ* are determined by grid search using 5 fold cross validation. However, in our case the grid search turned out to be computationally very intensive. Thus, we used 5% of the training dataset to determine the optimal parameters instead. The DisPredict output classes such as disordered or ordered residue, in terms of probability, is optimized by another round of 5-fold cross validation. Using the threshold value 0.5, the probabilities are converted into binary decision variables, where probability ranges 0.5 ≤ *range*_*d*_ ≤ 1.0 is considered as disordered and 0.0 ≤ *range*_*o*_ < 0.5 is considered as ordered. Fig 2 shows the detail paradigm of DisPredict.

We implemented our software in C++. The software is developed and tested on Linux platform. It is dependent on two external packages, namely PSI-BLAST [60] and NR database [27, 64], which are publicly available. DisPredict software is also available online at https://github.com/tamjidul/DisPredict_v1.0 with a user manual.

### 2.5 Performance Evaluation and Statistical Test Criteria

The performance of DisPredict is evaluated using the criteria followed in the past Critical Assessment of protein Structure Prediction (CASP) competitions [74–76]. The measures and procedures used in CASP experiments are comprehensive. The predictions are done in two levels:

1. Binary value, defining whether a residue is disorder or not (“+1” for disorder and “−1” for order) and
2. Real value, quantifying the probability of a residue being disorder (“≥ 0.5” for disorder and “< 0.5” for order).

#### Binary prediction evaluation

In binary (two-class) prediction of disorder, *TP* (True Positive) = number of correctly predicted disordered residues, *TN* (True Negative) = number of correctly predicted ordered residues, *FP* (False Positive) = number of incorrectly predicted disordered residues and *FN* (False Negative) = number of incorrectly predicted ordered residues. To determine the total number of correct prediction (both ordered and disordered), *N*_*correct*_ = *TP* + *TN* is calculated. Sensitivity *(SENS)* and specificity *(SPEC)* are two complementary statistical measures identifying the proportionate values of correct prediction of disordered (positive class) and ordered (negative class) residues, respectively.

$$\begin{array}{c} SENS=\frac{TP}{TP+FN}=\frac{TP}{N_{d}}\;\text{and}\;SPEC=\frac{TN}{TN+FP}=\frac{TN}{N_{o}} \end{array}$$

Here, *N*_*d*_ and *N*_*o*_ are the total number of disordered and ordered residues, respectively. As increment of one of these measures (SENS and SPEC) usually leads towards the decrement of another measure, neither of these two measures is a suitable indicator of performance for an imbalanced dataset. On the contrary, the balanced accuracy (ACC), weighted score (*S*_*w*_) and Mathews correlation coefficient (*MCC*) are the measures that take all four components of prediction quality (TP, TN, FP and FN) into account and thus can be regarded as more important indicators.

$$\begin{array}{c} ACC=\frac{1}{2}\left(\frac{TP}{TP+FN}+\frac{TN}{TN+FP}\right)\\ MCC=\frac{\left(TP\times TN)-(FP\times FN\right)}{\sqrt{\left(TP+FP)(TP+FN)(TN+FP)(TN+FN\right)}}\\ S_{w}=\frac{w_{d}\times TP-w_{o}\times FP+w_{o}\times TN-w_{d}\times FN}{w_{d}\times N_{d}+w_{o}\times N_{o}} \end{array}$$

where, *w*_*d*_ is the weight for *N*_*d*_ = percentage of ordered residues $=\frac{N_{o}}{N_{o}+N_{d}}$ and *w*_*o*_ is the weight for *N*_*o*_ = percentage of disordered residues $=\frac{N_{d}}{N_{o}+N_{d}}$ [75]. The *S*_*w*_ measure includes weight to address the imbalance in the ratio of ordered and disordered residues and rewards correct disorder classification over correct classification of ordered residues, which is later found to have a linear relationship with *ACC* (*S*_*w*_ = 2 × *ACC* − 1) [77]. Since both of these measures (ACC and *S*_*w*_) have been used in CASP assessment, we have also included both of them in our paper instead of just one. *MCC* score, another measure that accounts for all four parameters of the prediction quality, is the most reasonable and consistent measure for disorder prediction assessment because of not being favorable to over prediction of any class (order/disorder). *MCC* and *S*_*w*_ scores vary from −1 to 1, where −1 and 1 represent perfect misclassification and classification, respectively with a random classification scoring by 0. More recently, precision ($PPV=\frac{TP}{TP+FP}$) has been appeared as a good measure for binary disorder prediction as it is totally insensitive to the prediction of the dominant class (i.e., here the order state), is therefore computed to evaluate DisPredict. As the prediction becomes better, the values of these metrics also get higher.

We calculated Mean Absolute Error $(MAE)=\frac{\sum_{i=1}^{n}|c_{d}^{a}(i)-c_{d}^{p}(i)|}{n}$ to quantify the error of disorder prediction in content level. Here, *n* is the total number of protein chains, and $c_{d}^{a}(i)$ and $c_{d}^{p}(i)$ are the actual and predicted disorder content (fraction of disordered residues) for the *i* ^*th*^ protein chain, respectively. The lower value of MAE corresponds to better prediction.

#### Evaluation of predicted probability

The SVM model of DisPredict generates a predicted probability value for each residue which signifies the disorder confidence of that residue. This probability value is then binarized using a threshold of 0.5 to generate class annotation. If the probability is greater than or equal to 0.5, the predicted class is ‘disorder’ and if the probability is less than 0.5, the predicted class is ‘order’. Assessment of the predicted probability by a DisPredict is performed by receiver operating characteristic (*ROC*) curve, which depicts the correlation between the true positive rate (*TPR* or, *SENS*) and false positive rate (*FPR* = 1—*SPEC*) for a probability threshold. The area under the ROC curve (*AUC*) quantifies the predictive quality of a classifier, where the AUC value equal to 1 indicates a perfect prediction and 0.5 corresponds to a random prediction. Moreover, 95% confidence interval (CI) for the AUC score is evaluated using DeLong’s [78] variance estimated by bootstrapping. The evaluation of AUC and CI are performed using the statistical R package with the pROC [79] library.

## 3 Test Procedures and Results

### 3.1 Performance of 10-Fold Cross Validation

**Fig 3:**
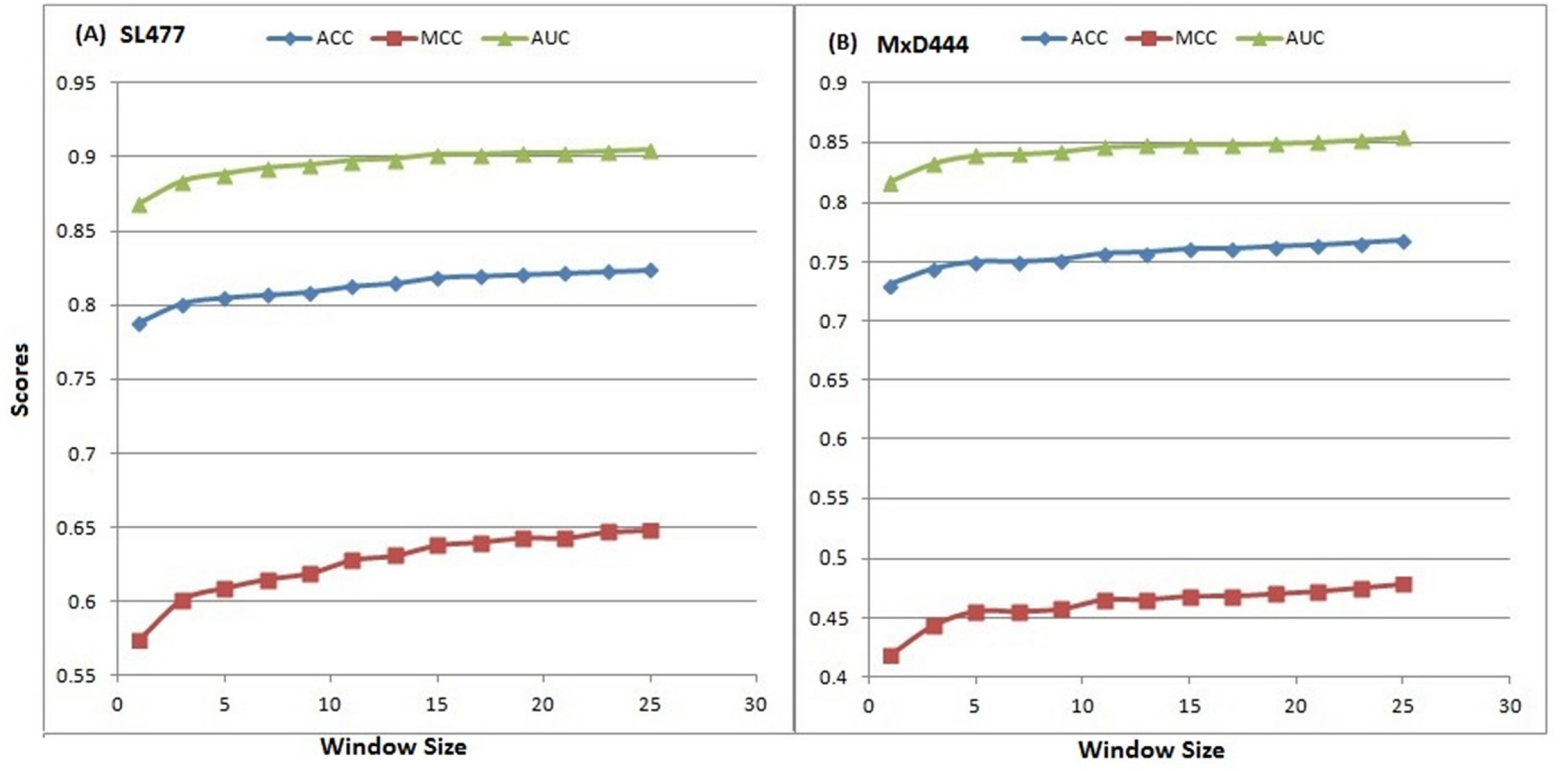
Increase of performance in 10-fold cross validation (default parameter) according to ACC, MCC and AUC scores with the increase of window size for (A) SL477 and (B) MxD444 dataset. The x-axis and y-axis represent the window sizes and scores, respectively.

We evaluated the 10-fold cross validation performance of DisPredict separately on SL477 and MxD444 dataset. Regarding the optimum selection of the window size, we ran cross validation individually for 13 different windows, shown in Table 2, for both of the SL477 and MxD444 dataset with default parameters for SVM. The best result for window size 25 was found with ACC, MCC and AUC values equal to 0.82, 0.65 and 0.91, respectively for SL477 dataset, whereas for MxD444 dataset the values are 0.77, 0.48 and 0.85, respectively. The gradual increase in performance becomes a plateau as window goes higher above size 23 (Fig 3). Table 2 also depicts the inverse relationship between SENS and SPEC scores with increasing window size for MxD444 dataset. The best SENS (0.74) is achieved by window size 25 while the best SPEC (0.81) is achieved at window size 5. Overall, the consistent increment in balanced accuracy (ACC) and PPV prove our methodology to be well balanced.

**Table 2:** 10-fold Cross Validation Performance of DisPredict (Default Parameter).

*W*_*size*_	TP	TN	FP	FN	*N*_*correct*_(*Residue*_*total*_)^1^	SENS	SPEC	ACC	*S*_*w*_	PPV	MCC	AUC [95% CI]^2^
SL477 Dataset												
1	4440	5804	1469	1260	10244 (12973)	0.779	0.798	0.788	0.577	0.751	0.574	0.869 [0.862, 0.876]
3	4467	5954	1319	1233	10421 (12973)	0.784	0.819	0.801	0.602	0.772	0.601	0.884 [0.877, 0.890]
5	4457	6020	1253	1243	10477 (12973)	0.782	0.828	0.805	0.609	0.781	0.609	0.889 [0.882, 0.896]
7	4441	6076	1197	1259	10517 (12973)	0.779	0.835	0.807	0.614	0.787	0.615	0.893 [0.886, 0.899]
9	4457	6086	1187	1243	10543 (12973)	0.782	0.837	0.809	0.618	0.789	0.619	0.895 [0.888, 0.902]
11	4483	6113	1160	1217	10596 (12973)	0.786	0.841	0.813	0.627	0.794	0.628	0.898 [0.891, 0.905]
13	4502	6114	1159	1198	10616 (12973)	0.790	0.841	0.815	0.630	0.795	0.631	0.899 [0.892, 0.905]
15	4513	6150	1123	1187	10663 (12973)	0.792	0.845	0.819	0.637	0.801	0.638	0.902 [0.896, 0.909]
17	4540	6133	1140	1160	10673 (12973)	0.796	0.843	0.820	0.640	0.799	0.640	0.902 [0.895, 0.902]
19	4545	6148	1125	1155	10693 (12973)	0.797	0.845	0.821	0.643	0.802	0.643	0.903 [0.896, 0.910]
21	4548	6148	1125	1152	10696 (12973)	0.798	0.845	0.822	0.643	0.802	0.643	0.903 [0.896, 0.910]
23	4555	6167	1106	1145	10722 (12973)	0.800	0.847	0.823	0.647	0.804	0.647	0.904 [0.898, 0.911]
25	4564	6164	1109	1136	**10728** (12973)	**0.801**	**0.847**	**0.824**	**0.648**	**0.804**	**0.648**	**0.905 [0.898, 0.911]**
MxD444 Dataset												
1	3284	13093	3397	1632	16377 (21406)	0.668	0.793	0.731	0.462	0.491	0.419	0.817 [0.810, 0.825]
3	3369	13241	3249	1547	16610 (21406)	0.685	0.803	0.744	0.488	0.509	0.444	0.832 [0.826, 0.840]
5	3410	13302	3188	1506	16712 (21406)	0.694	**0.807**	0.750	0.500	0.516	0.456	0.839 [0.833, 0.847]
7	3419	13275	3215	1497	16694 (21406)	0.695	0.804	0.750	0.501	0.515	0.455	0.840 [0.833, 0.847]
9	3446	13253	3237	1470	16699 (21406)	0.700	0.805	0.752	0.505	0.516	0.458	0.842 [0.834, 0.849]
11	3503	13232	3258	1413	16735 (21406)	0.712	0.802	0.757	0.515	0.517	0.466	0.846 [0.839, 0.853]
13	3523	13188	3302	1393	16711 (21406)	0.717	0.800	0.758	0.516	0.516	0.466	0.847 [0.839, 0.853]
15	3564	13145	3345	1352	16709 (21406)	0.725	0.797	0.761	0.522	0.515	0.469	0.848 [0.842, 0.855]
17	3578	13097	3393	1338	16675 (21406)	0.728	0.794	0.761	0.522	0.513	0.469	0.848 [0.841, 0.855]
19	3607	13068	3422	1309	16675 (21406)	0.734	0.792	0.763	0.526	0.513	0.471	0.849 [0.842, 0.856]
21	3613	13078	3412	1303	16691 (21406)	0.735	0.793	0.764	0.528	0.514	0.473	0.850 [0.843, 0.857]
23	3640	13059	3431	1276	16699 (21406)	0.740	0.792	0.766	0.532	0.515	0.476	0.851 [0.845, 0.859]
25	3658	13064	3426	1258	**16722** (21406)	**0.744**	0.792	**0.768**	**0.536**	**0.517**	**0.479**	**0.852 [0.847, 0.861]**

W_*size*_ indicates the size of sliding window.

Best values of each metric are marked in bold for each dataset separately.

^1^ N_*correct*_ is reported with total number of residues (Residue_*total*_) to be predicted in parentheses. Both of the counts correspond to one subset (fold) of the full dataset which is generated for performing cross validation.

^2^ For AUC, the values within bracket indicate 95% confidence interval with 2000 stratified bootstrap replicas.

As the window size continues to increase, the rate of increase in scores becomes slow. Increase of scores is ≤ 0.001, as the windows size grows from 23 to 25 for SL477 dataset and ≤ 0.004 for MxD444 dataset, respectively.

Note that, this preliminary extensive analysis of performance with multiple window sizes is done without selection of optimal parameters for SVM. For a specific window size (*W*_*size*_) and total number of residues (*Residue*_*total*_) in a dataset, we have a feature matrix of dimension, *Residue*_*total*_ × (*W*_*size*_ × 56). Therefore, the increase in window size leads towards the increase in the dimensions of the feature space, which in turn makes the time expensive grid search for parameters slower. To trade off between performance with optimization and time complexity of parameter selection along with model generation, we determined the optimal values of parameters with a 5% randomly selected subset of residues from training dataset for 3 window sizes (15, 21 and 25). The optimal parameters (*C* and *γ*) found from grid search are reported in Table 3. Furthermore, we inserted repeated disordered residue information only in case of training to balance the dataset as the support vector points for the less dominant class may not be sufficient to determine the optimal SVM margin. Specifically, duplicates (2 times for SL477 dataset and 3 times for MxD444 dataset) of disorder samples were provided during generation of predictor model. However, in case of testing, no repeated information was inserted. Table 4 illustrates the detail of the cross validation results with optimized parameters for 3 different window sizes.

**Table 3:** Optimized Parameters used to build DisPredict Models.

	SL477 Dataset		MxD444 Dataset	
*W*_*size*_	*C*	*γ*	*C*	*γ*
15	8.0	0.001953125	8.0	0.0312500
21	2.0	0.007812500	2.0	0.0078125
25	0.5	0.007812500	2.0	0.0078125

*C* is the soft penalty parameter to handle overlapped class.

*γ* is the parameter for radial basis kernel for SVM.

**Table 4:** 10-fold Cross Validation Performance of DisPredict (Optimized Parameter).

*W*_*size*_	TP	TN	FP	FN	*N*_*correct*_(*Residue*_*total*_)^1^	SENS	SPEC	ACC	*S*_*w*_	PPV	MCC	AUC [95% CI]^2^
SL477 Dataset												
15	4655	6056	1217	1045	10711 (12973)	**0.817**	0.833	0.825	0.649	0.793	0.647	0.898 [0.890, 0.904]
21*	4617	6271	1002	1083	**10888** (12973)	0.810	**0.862**	**0.836**	**0.672**	**0.822**	**0.673**	**0.956 [0.950, 0.963]**
25	4624	6234	1039	1076	10858 (12973)	0.810	0.857	0.834	0.668	0.816	0.669	0.911 [0.904, 0.917]
MxD444 Dataset												
15	2590	15590	900	2326	18180 (21406)	0.527	**0.945**	0.736	0.472	**0.742**	0.538	0.838 [0.831, 0.845]
21*	3480	14890	1600	1436	**18370** (21406)	**0.708**	0.903	**0.805**	**0.611**	0.685	**0.600**	**0.853 [0.847, 0.859]**
25	3367	3367	1635	1549	18222 (21406)	0.685	0.901	0.793	0.586	0.673	0.582	0.850 [0.843, 0.858]

W_*size*_ indicates the size of sliding window and * mark represents window size with overall optimal (best) performance.

Best values of each metric are marked in bold for each dataset separately.

^1^ N_*correct*_ is reported with total number of residues (Residue_*total*_) to be predicted in parentheses. Both of the counts correspond to one subset (fold) of the full dataset which is generated for performing cross validation.

^2^ For AUC, the values within bracket indicate 95% confidence interval with 2000 stratified bootstrap replicas.

The improvement of performance with optimized parameters over non-optimized one was significant. To compare, for SL477 dataset (window size 21), FP and FN values are reduced to 1,002 and 1,083 from 1,125 and 1,152 due to optimization. In case of MxD dataset (window size 21), the FN value is increased by 133 residues. However, the FP value is also decreased by 1,812 residues which maintains the overall increase in the total number of correctly predicted residues from 16,691 to 18,370. The improvement of prediction, both in terms of increased correct classification and decreased misclassification, is also visible from both the sensitivity and specificity scores. For window size 21, the values of *S*_*w*_, precision and MCC are improved by 4.5%, 2.5% and 4.5% respectively due to optimized training on SL477 dataset. At the same time, for MxD444 dataset, these progresses are 15.7%, 33.3% and 26.8% respectively. Note that, this significant improvement in MCC strongly supports our method’s capability in handling the imbalance ratio of ordered and disordered residues. Further, the AUC score is also increased by 4.4% and 0.4% as the result of optimization for SL477 and MxD444 dataset, respectively. A comparative analysis of Table 2 and Table 4 also shows that optimized DisPredict model with window size 21 outperforms all the other models of its own kind. Thus we select 21 as the optimal window size for our proposed DisPredict. Furthermore, to understand the relevance of the new features (MGs and BGs) with protein disorder, we separately evaluated optimized DisPredict’s performance without monograms and bigrams. We performed 10-fold cross validation on SL477 dataset with the optimal window size 21 and optimal parameters of SVM as reported in Table 3 for SL477 dataset with window size 21. The result of this experiment in terms of ACC, MCC and *S*_*w*_ score are 0.810, 0.651 and 0.621, respectively. The comparison of these scores excluding MGs and BGs with those of including MGs and BGs (reported in Table 4 for SL477 dataset) shows that involvement of MGs and BGs along with PSSM leads to a further increase in binary prediction accuracy in terms of 3.2% improved ACC (0.810 to 0.836), 3.8% improved MCC (0.651 to 0.673) and 8.2% improved *S*_*w*_ score (0.621 to 0.672).

To uniformly distribute the residues into ten subsets for cross validation, we applied modular arithmetic operation to split the dataset in residue level. As the residues are already included within the neighboring information based on the window, they are detachable from their original sequence. However, this inclusion of residue information within window may yield overlap of information between training and test sets in case of residue level splitting of dataset for cross validation. We analyzed the probability of this residual overlap between training and test sets. Let, there are *N* sequences in the dataset and the expected length of the sequence is ℒ. Then, the possibility of picking two residues for training and test subsets of 10 fold cross validation which belongs to same sequence is $(\frac{1}{\frac{9N}{10}}\times \frac{1}{\frac{N}{10}})=\frac{100}{9N^{2}}$. Since the expected length of a sequence is ℒ, the chance of training and test overlap for a specific window size (*W*_*size*_) is $\frac{W_{size}-1}{ℒ}$. Altogether, the probability of a train and test residue overlap from the same sequence is $(\frac{100}{9N^{2}}\times \frac{W_{size}-1}{ℒ})=(\frac{100}{9})\frac{W_{size}-1}{N^{2}ℒ}$. For SL477 dataset with *N* = 477, approximate ℒ=400 and *W*_*size*_ = 21, the probability of the overlap is 2.44 × 10^−06^, which is significantly low and thus can be safely ignored. Further, we reevaluated DisPredict’s 10 fold cross validation performance with sequence level sampling by modular operation for SL477 dataset to generate training and test subsets. Table 5 quantifies the difference in performance between residue level and state of the art practice of sequence level splitting of dataset for cross validation with window size 21 and default parameters for SVM. It justifies that DisPredict’s performance remains consistent without any significant over prediction in terms of all the metrics.

**Table 5:** DisPredict’s cross validation performance with residue level and sequence level splitting of SL477 dataset.

Splitting Method	SENS	SPEC	ACC	*S*_*w*_	PPV	MCC	AUC [95% CI]
Residue Level	0.798	0.845	0.822	0.643	0.802	0.643	0.903 [0.896, 0.910]
Sequence Level	0.784	0.844	0.814	0.628	0.793	0.627	0.892 [0.886, 0.898]

Default values of *C* and *γ* are applied for SVM.

Window size 21 is used.

### 3.2 Evaluation of Independent Training and Testing

With optimized parameters and balanced dataset, we carried out independent training on SL477 and MxD444 datasets followed by testing the resulting predictor model with MxD134 and SL171 dataset, respectively. Note that, these independent test datasets (MxD134 and SL171) were generated at low sequence identity (10%) with the corresponding training datasets (SL477 and MxD444). The consistent results of these two tests done through cross validation and independent test confirm the usage of robust technique and effective feature set in DisPredict as well as training efficacy avoiding possible over-fittings. Table 6 further illustrates the results of these tests, where we reported the average of the scores computed for equally divided 10 subsets of the full dataset along with the corresponding standard deviation (STDEV). Table 6 reveals that training by SL477 dataset gives consistent performance regardless of test datasets and test procedures (cross validation or independent test) in terms of ACC: 0.836, 0.833 and *S*_*w*_: 0.672, 0.667. These consistencies are also evident in case of training with MxD444 dataset while tested by different datasets and the evaluations are, ACC: 0.805, 0.789 and *S*_*w*_: 0.611, 0.577. We calculated the Mean Absolute Error (MAE) which is also reported along with its corresponding STDEV from mean. The score indicates that the error does not increase from cross validation to independent test as the test-results were robust.

**Table 6:** Performance Comparison of Cross Validation and Independent Tests.

Model Evaluation Procedure^1^	SENS (STDEV)	SPEC (STDEV)	ACC (STDEV)	*S*_*w*_ (STDEV)	PPV (STDEV)	MCC (STDEV)	AUC (STDEV)	MAE (STDEV)
10-fold cross validation on SL477	0.810 (0.004)	0.862 (0.001)	0.836 (0.002)	0.672 (0.005)	0.822 (0.002)	0.673 (0.004)	0.956 (0.007)	0.032 (0.002)
Train by SL477, Test on MxD134	0.744 (0.002)	0.923 (0.002)	0.833 (0.002)	0.667 (0.003)	0.574 (0.002)	0.598 (0.004)	0.906 (0.001)	0.023 (0.001)
10-fold cross validation on MxD444	0.708 (0.006)	0.903 (0.001)	0.805 (0.003)	0.611 (0.006)	0.685 (0.002)	0.600 (0.004)	0.853 (0.007)	0.208 (0.001)
Train by MxD444, Test on SL171	0.718 (0.003)	0.860 (0.001)	0.789 (0.001)	0.577 (0.003)	0.748 (0.003)	0.583 (0.002)	0.872 (0.007)	0.151 (0.001)

^1^ All the evaluations are carried out using a sliding window of length 21 and optimized parameters for SVM.

**Fig 4:**
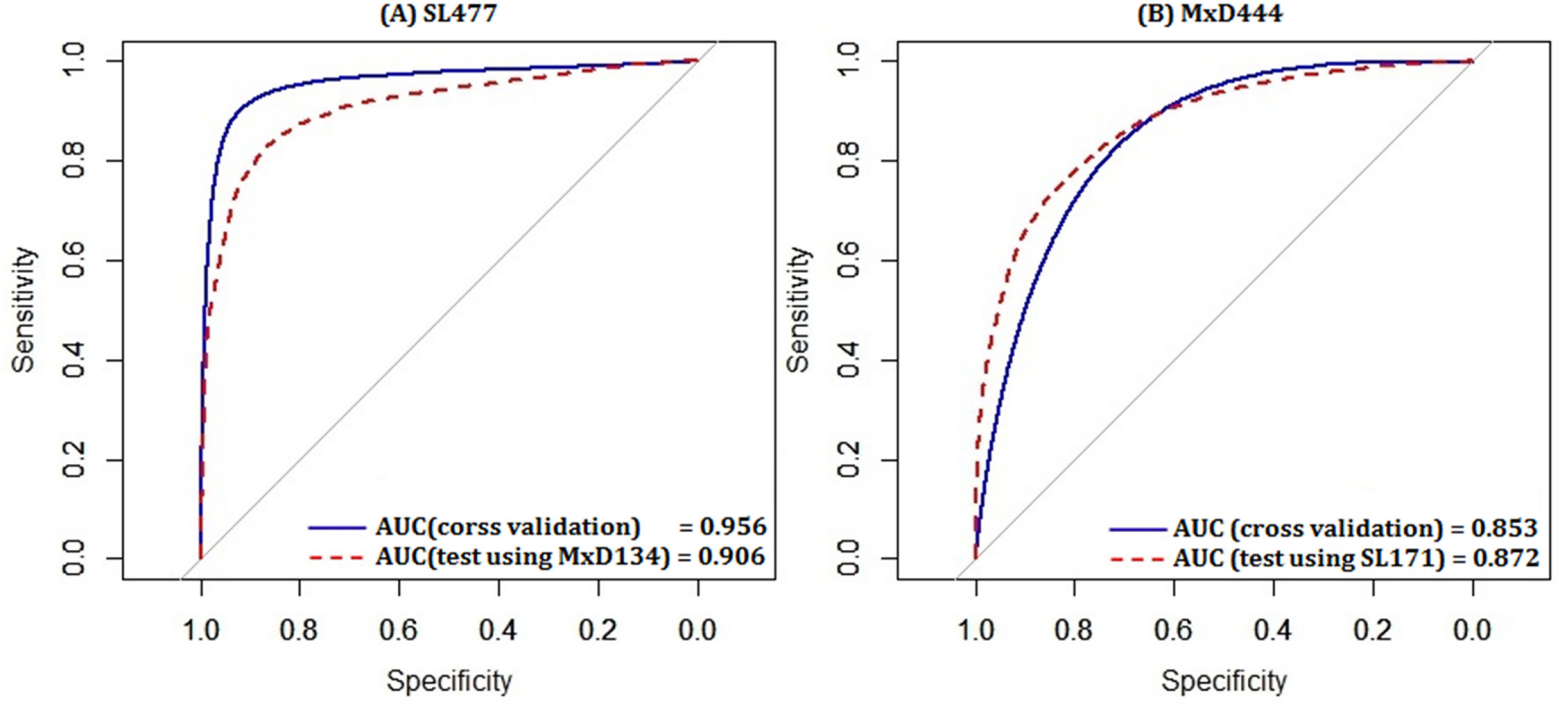
ROC curves given by DisPredict for the probability prediction per residue while the training is performed with (A) SL477 and (B) MxD444 dataset. In each figure, the solid (*blue*) curve corresponds to the cross validation test on the same dataset and the dotted (*red*) curve corresponds to the independent test. The AUC values given in each figure correspond to the values in Table 6. The x-axis and y-axis show the Specificity and Sensitivity, respectively.

To analyze the probability prediction, the ROC curves given by DisPredict are plotted in Fig 4 in continuous scale between 0.0 and 1.0. In each figure, two ROCs are plotted keeping the training dataset same with varying test datasets and evaluation procedure. Finally, we reported the AUC values which are found consistent for cross validation and independent test indicating our predictor’s capability to avoid over-fitting.

### 3.3 Comparison with Existing Predictors

The performance of DisPredict is compared with the state-of-the-art disorder predictors, MFDp [56] and SPINE-D [47]. To remain fair while comparing DisPredict with each of the above two predictors, we train DisPredict separately with respective datasets and compare with each of them separately. Thus, DisPredict is compared with MFDp based on dataset MxD444, while dataset SL477 is used to compare DisPredict with SPINE-D (Table 7).

**Table 7:** Comparative predictive quality of DisPredict with MFDp on MxD444 dataset and SPINE-D on SL477 dataset.

Method	SENS	SPEC	ACC	*S*_*w*_	MCC	AUC
DisPredict^2^	0.71	**0.90**	**0.80**	**0.61**	**0.60**	**0.85**
MFDp^1^	**0.76**	0.75	0.75	0.51	0.44	0.81
DisPredict^3^	**0.81**	**0.86**	**0.84**	**0.67**	**0.67**	**0.96**
SPINE-D^4^	0.77	0.85	0.81	0.62	0.63	0.87

^1^ 5-fold cross validation performance of MFDp on MxD dataset of 514 protein chains [56].

^2^ 10-fold cross validation performance of DisPredict on MxD444 which is a subset of 444 chains out of 514 chains with no X-tag.

^3^ 10-fold cross validation performance of DisPredict on SL477.

^4^ 10-fold cross validation performance of SPINE-D [47] on SL477.

In particular, MFDp [56] is a meta predictor that combines the predictions from three disorder predictors (DISOPRED2 [32], IUPred [50] and DISOclust [53]). Further, MFDp combines the outputs from three SVMs with linear kernel using a threshold of 0.37, used to output binary prediction. In contrast, we utilized single SVM with RBF kernel and optimized parameters combined with a comprehensive set of features to develop the standalone predictor. However, the performance of MFDp in Table 7 is of 5-fold cross validation whereas DisPredict is evaluated by 10-fold cross validation and hence to be considered reliable rather than over-fitted by chance. In terms of MCC, DisPredict improved significantly, which is 36.36% better than MFDp. The improvement in *S*_*w*_ score is also 19.6%. DisPredict showed lower sensitivity (7%) than MFDp while at the same time improved specificity by 20%, which in turn improved the balanced accuracy by 6.67%. Moreover, DisPredict outperformed MFDp in AUC score by 1.29% which is used to assess the probability based prediction.

The other state of the art predictor, SPINE-D [47] utilizes ANN technique which was at first developed to output three state prediction and later reduced into two state predictor of ordered and disordered residues. SPINE-D employs a disorder probability threshold of 0.06 that was optimized to achieve maximum S_*w*_ score. On the contrary, DisPredict is a SVM based two state disorder predictor using a more meaningful threshold for two-class classification of value 0.5. DisPredict outperformed SPINE-D in terms of sensitivity as well as specificity by 5.19% and 1.18% respectively which leads to 3.7% improvement in overall accuracy. DisPredict also outperformed SPINE-D in terms of S_*w*_, MCC and AUC by 8.06%, 6.34% and 10.34% respectively.

**Fig 5:**
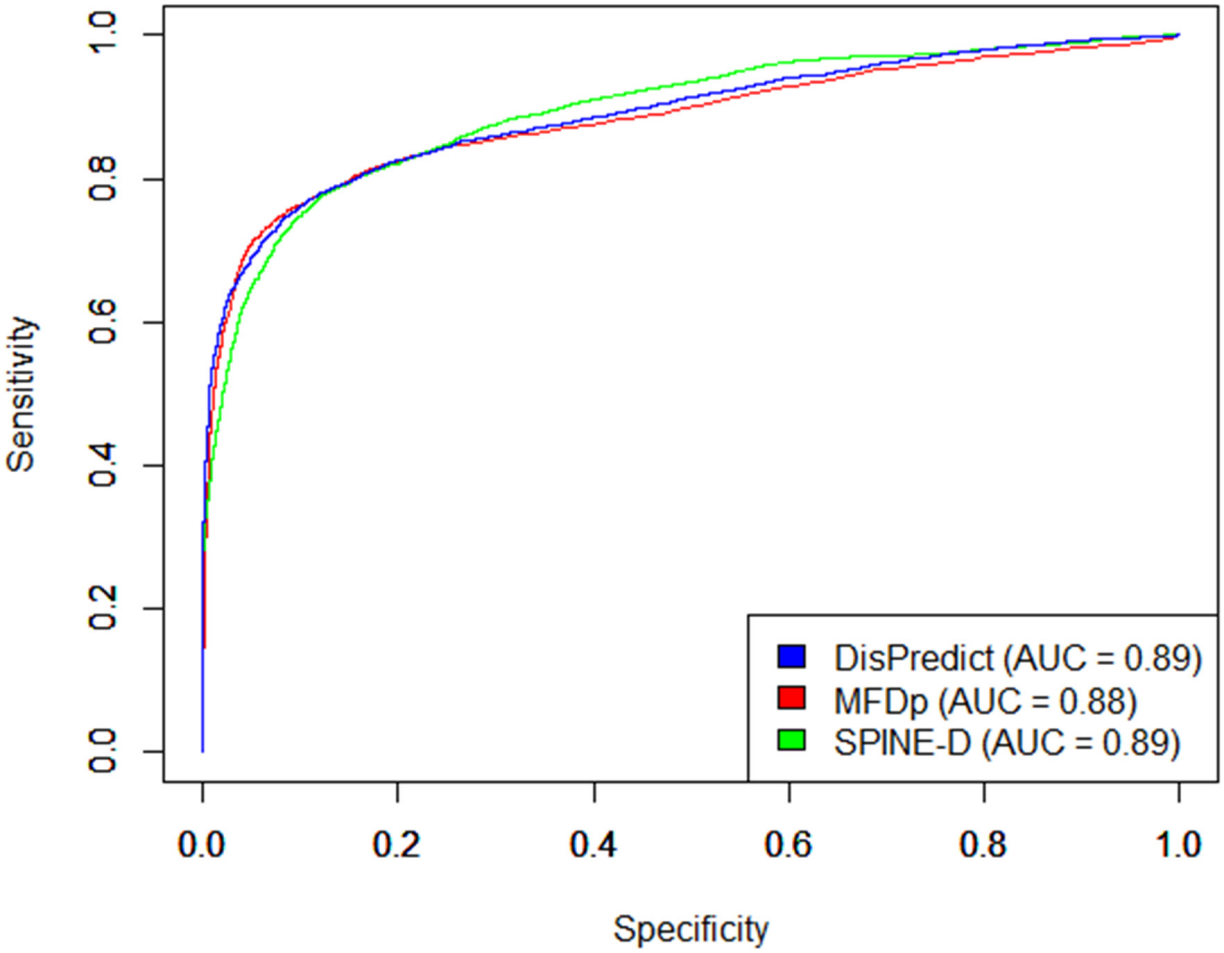
ROC curves for disorder prediction on DD73 dataset given by DisPredict(*blue*), SPINE-D(*green*) and MFDp(*red*). The AUC values shown in the figure correspond to the values in Table 8. The x-axis and y-axis show the Specificity and Sensitivity, respectively.

**Fig 6:**
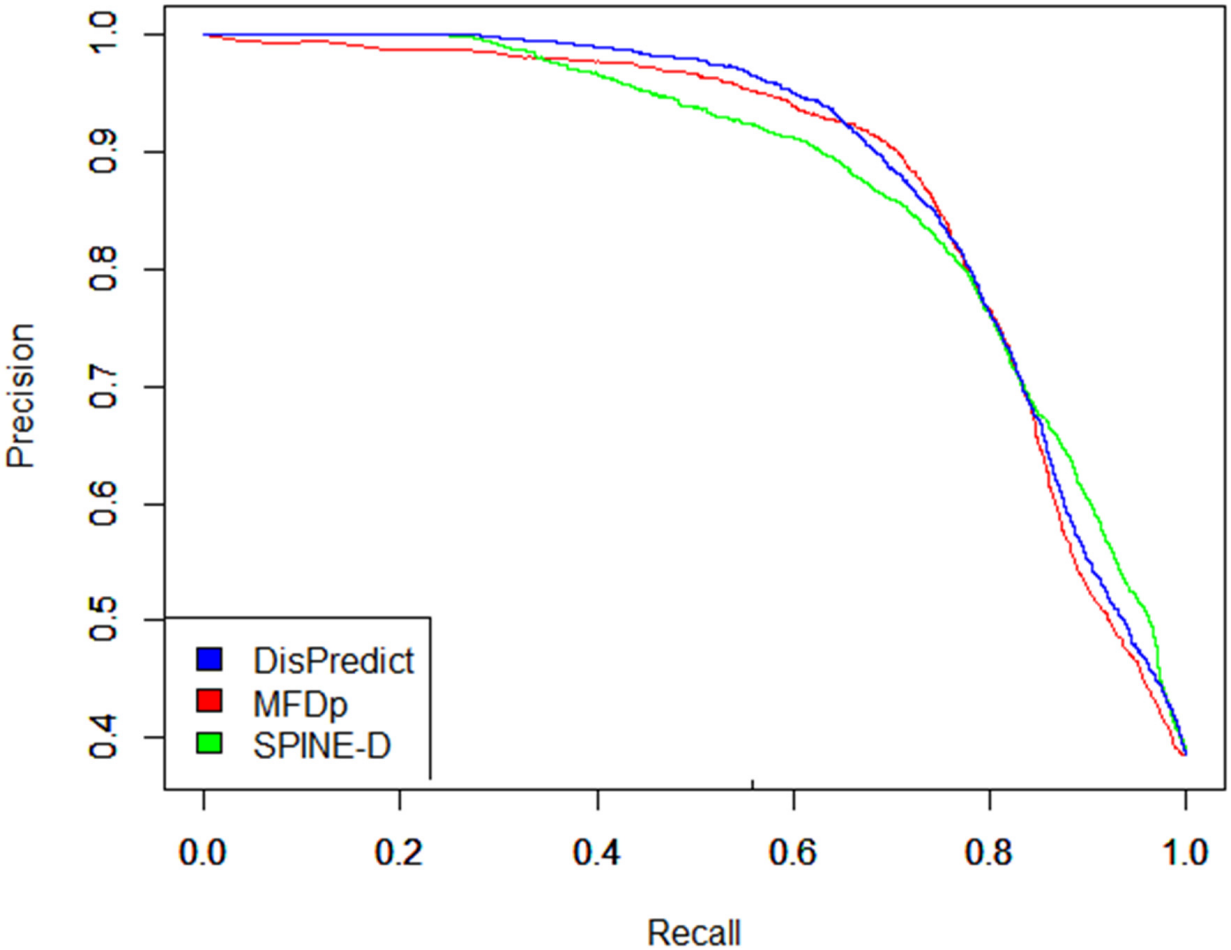
Precision-Recall curves for disorder prediction on DD73 dataset given by DisPredict(*blue*), SPINE-D(*green*) and MFDp(*red*). The x-axis and y-axis show the Recall(Sensitivity) and Precision (PPV), respectively.

In addition to the comparison on cross validation test, we evaluated DisPredict, SPINE-D [47] and MFDp [56] on independent DD73 dataset. The comparison among these three methods is illustrated in Table 8. It shows that DisPredict gives better performance among three predictors except in case of sensitivity. DisPredict yielded 2.63% lower sensitivity than that of SPINE-D [47], whereas DisPredict gave 4.25% higher specificity than that of SPINE-D [47]. Table 8 also shows that DisPredict outperformed SPINE-D [47] and MFDp [56] in terms of MCC by 3.76% and 0.76%, respectively. At the same time, DisPredict gave 1.26% and 5.36% improved precision (PPV) than MFDp [56] and SPINE-D [47], respectively. However, DisPredict resulted slightly lower sensitivity than those of SPINE-D [47] and MFDp [56]. At the same time, both SPINE-D [47] and MFDp [56] gave lower specificity than that of DisPredict. Figs 5 and 6 compare the ROC curves and precision-recall curves, respectively, given by DisPredict, SPINE-D [47] and MFDp [56]. Fig 5 shows that the ROC curves given by the three predictors are comparative. At the same time, the precision-recall curves (Fig 6) depicts that DisPredict achieves consistently higher precision upto less than 65% sensitivity (recall).

**Table 8:** Performane comparison among DisPredict, SPINE-D and MFDp on independent DD73 dataset.

Predictor	SENS	SPEC	ACC	*S*_*w*_	PPV	MCC	AUC [95% CI]
DisPredict*	0.775	**0.883**	**0.829**	**0.658**	**0.806**	**0.663**	**0.89 [0.88, 0.90]**
SPINE-D	**0.796**	0.847	0.822	0.644	0.765	0.639	**0.89 [0.88, 0.90]**
MFDp	0.780	0.875	0.828	0.656	0.796	0.658	0.88 [0.87, 0.89]

Best results are marked in bold.

* Window size = 21, *C* = 2.0 and *γ* = 0.0078125.

MFDp and SPINE-D have been established as the best disorder predictor among 8 and 11 existing disorder predictors [47, 56], respectively, covering different approaches in their relevant publication. In this article, our predictor is shown to be comparable with both of these methods. Therefore, DisPredict can be considered to be one of the finest disorder predictor and can be utilized to produce more reliable annotation of disorder versus order residues.

### 3.4 Case Studies: Characteristic Region and Protein Function

**Fig 7:**
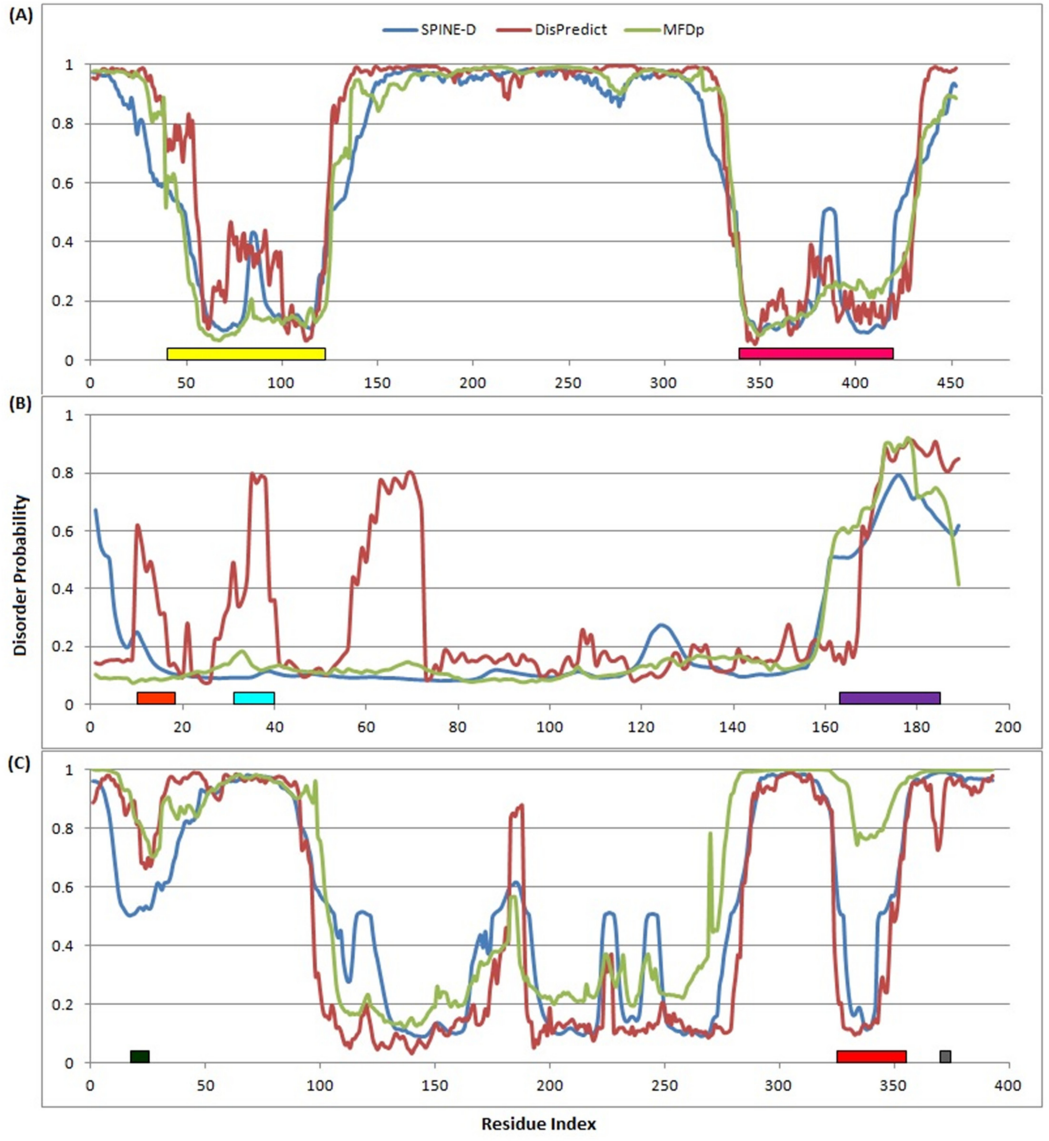
Disorder probability plot for (A) human ETV6 (P41212), (B) human KRAS (P01116) and (C) human p53 (P04637) proteins, given by DisPredict(*red*), SPINE-D (*blue*) and MFDp (*green*). In (P41212, A), the yellow (40 − 124 residues) and pink bar (339 − 420 residues) represent to the PNT domain [80] and ETS DNA binding region [81], respectively. In (P01116, B), the orange (10 − 17 residues), cyan (32 − 40 residues) and purple bar (166 − 185 residues) correspond to the GTP binding region [82], effector region and hypervariable region, respectively. In (P04637, C), the dark green (17 − 25 residues), red (325 − 356 residues) and gray bar (370 − 372 residues) highlight to the TADI motif [83], oligomer region and [KR]-[STA]-K binding motif, respectively.

Proteins with disordered regions are found to contain several regions of interest, such as self-stabilizing folded regions, DNA or, nucleotide binding regions, short (up to 20 amino acids) conserved regions of biological significance (known as motif), mediating regions for protein interaction with different partners etc. These characteristic regions undergo various conformational changes, gain structure and affect many important biological functions. We selected three proteins as cases (UniProt IDs: P41212, P01116 and P04637) with experimentally verified regions of interest to analyze per residue disorder confidence score assigned by DisPredict, SPINE-D and MFDp. Fig 7 illustrates the disorder probability of each residue with respect to residue index. P41212 (Fig 7(A)) is a human ETV6 protein for transcriptional repressor function, which is also involved in several kinds of leukemia and syndrome. For this protein, DisPredict and SPINE-D showed comparable performance in detecting the highly conserved region of PNT (pointed) domain [80] [residues 40 − 124] and ETS (E26 transformation-specific) DNA binding region [81] [residues 339 − 420], respectively, while MFDp outperformed both of them with relatively less noise. P01116 (Fig 7(B)) is a human KRAS protein with intrinsic GTPase activity (binds GDP/GTP [82]) and related to several diseases, such as gastric cancer (GASC), acute myelogenous leukemia (AML), cardiofaciocutaneous syndrome 2 (CFC2) etc. DisPredict could identify its GTP (guanosine triphosphate) binding region [residues 10 − 17] and effector region [residues 32 − 40] respectively, with close to cut-off (0.5) probabilities. Note that, these two regions are experimentally verified unstructured regions, which are strongly suggested as structured by both SPINE-D and MFDp. However, the C-terminal hypervariable region [residues 166 − 185] is consistently detected by all three of these predictors. P04637 corresponds to human p53 protein which acts as a tumor suppressor. Fig 7(C) illustrates that DisPredict and MFDp outperformed SPINE-D with relatively sharp detection of N-terminal TADI (transcriptional repression domain-I) motif [83] [residues 17 − 25]. On the other hand, DisPredict and SPINE-D outperformed MFDp in determining oligomerization domain [84] of residues 325 − 356. Fig 7(C) also shows that both SPINE-D and MFDp missed the very short, 3 residue (370 − 372) long [KR]-[STA]-K binding motif at C-terminal, while DisPredict detected it correctly. The overall comparison depicts that DisPredict’s performance is more biologically relevant with correct identification of these short regions. Therefore, it would be interesting to utilize DisPredict in a broader scope in near future.

## 4 Discussion

In this article, we proposed a canonical support vector machine which uses a RBF kernel and includes useful and advanced features for predicting disordered residues, called DisPredict. DisPredict not only generates the binary class annotation for ordered and disordered residues but also provides order-disorder probabilities that can be treated as the confidence level of the prediction too. The DisPredict outperforms other existing top performing predictors both in predicting binary annotation and probability. The competitive performance of DisPredict is mainly due to the use of a novel methodology that incorporates firstly, radial basis kernel function (RBF) that can implicitly map the feature space in infinite dimension, secondly and most importantly the optimization of the parameters and thirdly, the novel features monogram (MG) and bigram (BG) assisted in determining an optimal as well as effective class separating hyperplane.

This overall performance of DisPredict is also persuaded by the use of a comprehensive set of features that well captures the sequential (amino acid composition) and structural characterization of ordered and disordered residues or, proteins. We used SPINE X [65] to generate the secondary structure related fine features. The distinguishing property of our feature set in comparison with existing predictors is the inclusion of monogram (MG) and bigram (BG), computed from PSSM. When a region of a protein is evolutionary conserved in a fold, then all the proteins within that fold are likely to have a conserved group of MGs and BGs. As some intrinsic disordered regions are conserved, addition of these features provides important structural evolutionary characteristics. By determining the appropriate window size, we have also included the effect of optimal interactions due to the contacts among neighboring residues.

**Fig 8:**
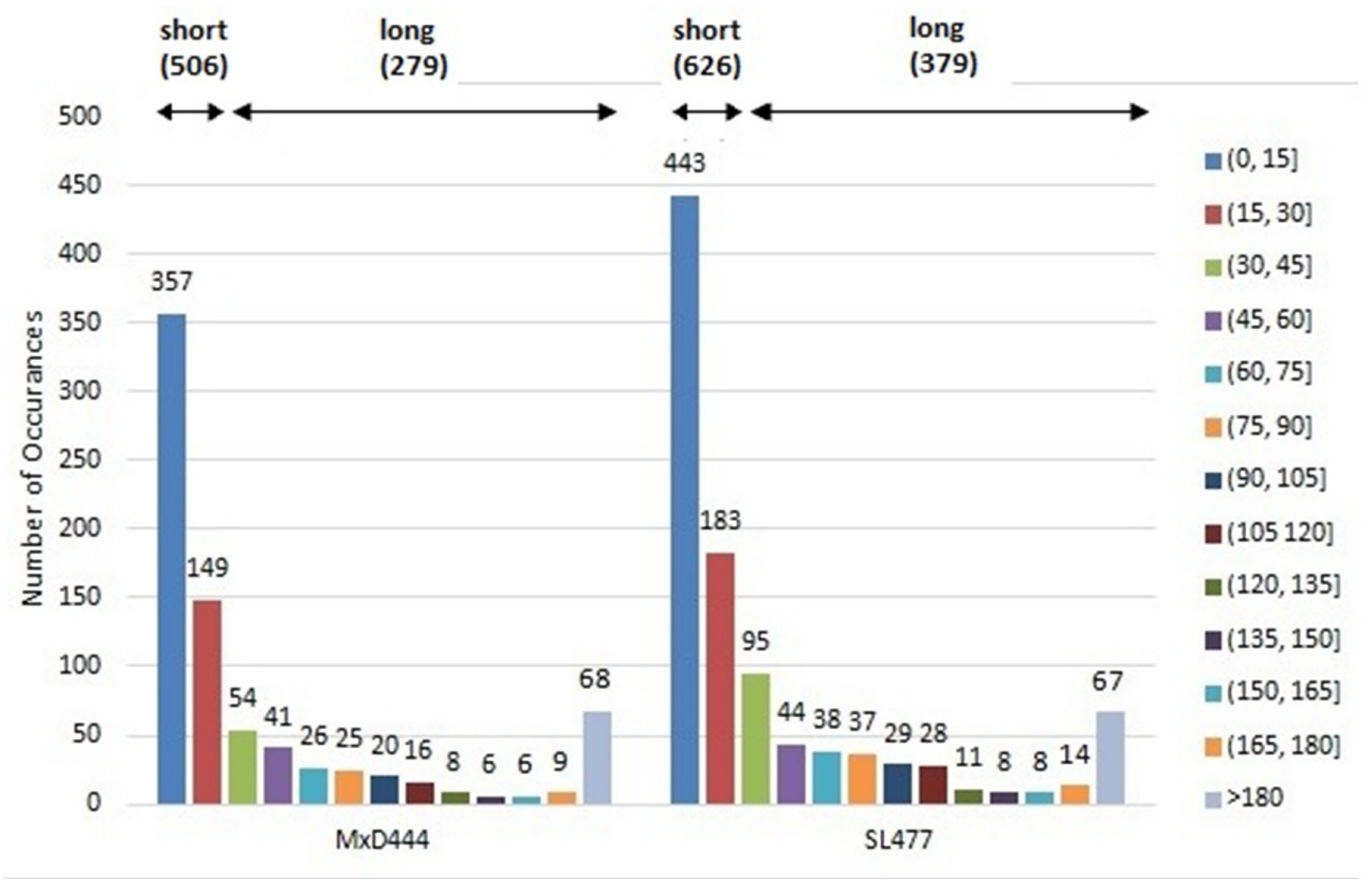
Distribution of disordered regions of different lengths in MxD444 (*left*) and SL477 (*right*) dataset. Legends are shown for different range of lengths (with interval size 15) and each bar is labeled with total number of occurrence of a disordered region of this specific length.

The robust performance of DisPredict is also justified by training and testing the predictor with multiple datasets: SL477, SL171 and MxD444, MxD134. The datasets used to train DisPredict encompass disorder annotation from several complementary sources (X-ray and NMR defined disorder from PDB and DisProt) as well as disorder region of various lengths. The SL dataset comprises of 81 full disordered proteins (IDPs) while the rest of the chains contain 928 disordered regions (IDRs). On the other hand, the MxD dataset is composed of 55 full disordered chains, 4 full ordered chains and 385 chains, sharing both structured and disordered regions, which include 730 disordered regions (IDRs). Furthermore, 70% of the IDRs included within partially disordered proteins are short (≤ 30 residues) and 30% of them are long (> 30 residues). This combination of several length disordered regions (Fig 8) included within training confirms the consistent performance of DisPredict for disordered regions of all sizes as well as different types of disordered residues.

**Fig 9:**
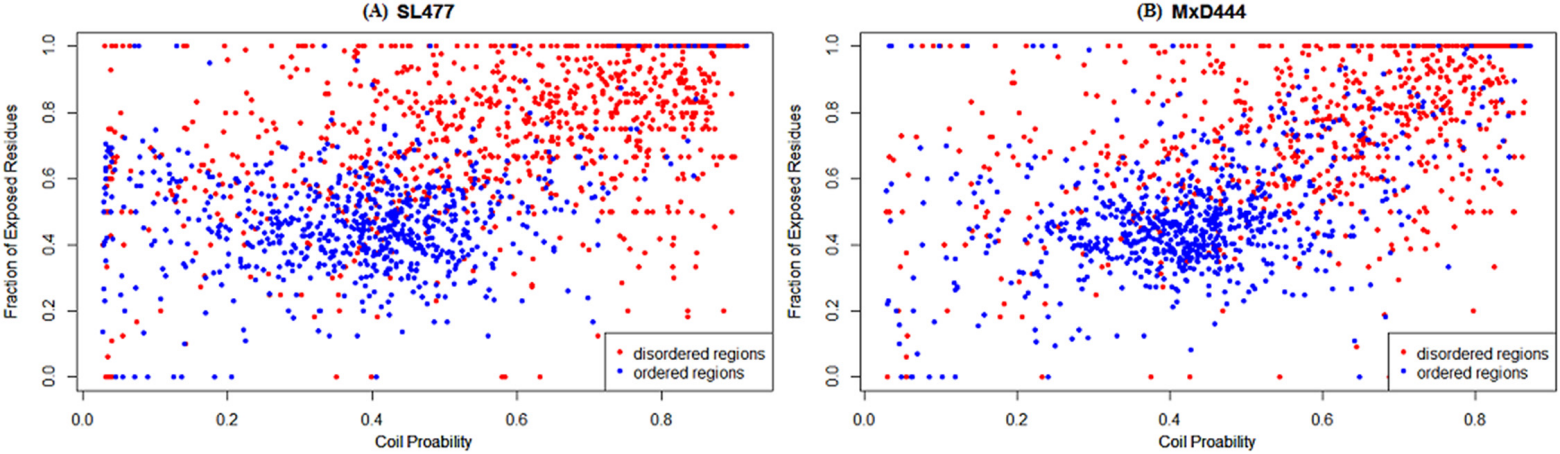
Correlation plot between structural characterizations of ordered (*blue*) and disordered (*red*) regions within (A) SL477 and (B) MxD444 dataset. The x-axis and y-axis correspond to the probability of having well defined secondary structure (in terms of probability being coil) and fraction of exposed residues of that region, respectively.

It is interesting to note that, regardless of cross validation or independent test, DisPredict’s performance is relatively better while it is trained on SL477 dataset than that of MxD444 (Table 6). To further insight into this discrepancy, we investigated the correlation of true annotation provided in the dataset with the actual structural characterization of disordered and ordered residues. Disordered residues are distinguished from ordered residues by low content of secondary structure [8, 28], therefore high probability of coil residues than helical or beta strand residues and disordered regions are likely to have large solvent accessible (exposed) area [55]. We represented the correlation of the fraction of secondary structure content and fraction of exposed residues for disordered and ordered regions of all length in Fig 9. We employed the predicted probability of each residue to be coil and predicted per residue solvent accessibility provided by SPINE-X [65] since all residues do not have defined coordinates (structure) to compute secondary structure and solvent accessibility.

We calculated the average coil probability (*P*_*coil*_) for each ordered or disordered region and computed the fraction of exposed residues with greater than 25% solvent accessibility (*F*_*exposed*_) of that region. In this analysis, we discarded 5 residues from N and C-terminal regions of each protein sequence as they are mostly found on the surface of a protein chain (not buried in the core) and more likely to be affected by the interaction with nearby structured protein, yielding to a highly flexible and dynamic conformation. The plots for both datasets show that the ordered regions are mostly concentrated in the portion with relatively low coil probability, 0.3 ≤ *P*_*coil*_ < 0.5 (high content of well defined helical or strand secondary structured residues) and low exposure, 0.2 ≤ *F*_*exposed*_ < 0.5. While on the contrary, the disorder regions are found abundant in the area of high coil probability, 0.5 ≤ *P*_*coil*_ ≤ 0.9 (low content of helical or strand secondary structured residues) and high exposure, 0.5 ≤ *F*_*exposed*_ ≤ 1.0. However, we found the intrinsic difference between these two datasets according to their annotation of residues as order and disorder. This difference is also evident from the top right location of the correlation plot, 0.6 ≤ *P*_*coil*_ ≤ 0.8 and 0.4 ≤ *P*_*coil*_ ≤ 0.9, designated for disordered regions. For SL477 dataset (Fig 9(A)), the number disordered regions are predominant over the number of ordered regions in this top right location of disordered regions in the plot. In contrast, the same location of the plot is overlapped by both ordered and disordered regions in case of MxD444. We further quantified the difference as 13% of the data in MxD444’s ordered set are more likely to be coil as well as highly exposed while 6% of the data in SL477’s ordered set are exposed as well as coil. This higher proportion of misleading annotation in MxD444 dataset contributes relatively lower signal to noise ratio (SNR) of 87/13 compared to 94/6 for SL477 which is the most compelling reason of the better performance of DisPredict in case of training dataset SL477 over MxD444. As the prediction produced by DisPredict is well capable of detecting such discrepancies in the native annotation of the datasets, it can be utilized as a reliable source of correct annotation of the ordered and disordered residues. We should also focus that, a similar proportion of 11% and 13% of the disordered data are also mixed with the ordered residues in the low coil probability region of the plot for both MxD444 and SL477 dataset, respectively.

**Fig 10:**
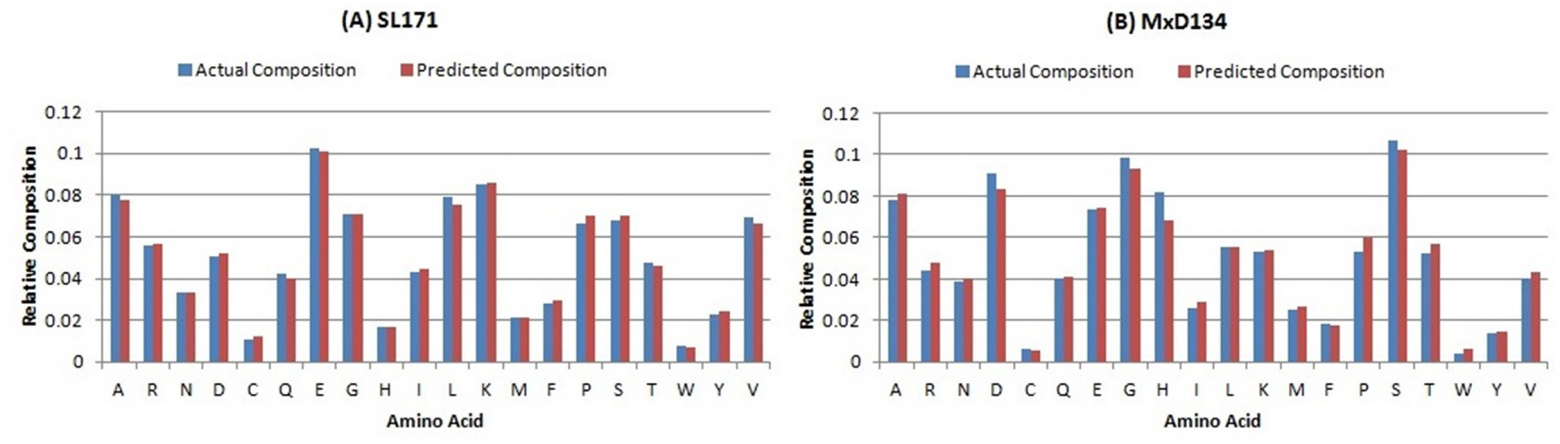
Percentage of amino acid type residues in actual composition (*blue, or left adjacent bar*) and predicted composition (*red, or right adjacent bar*) of (A) SL171 and (B) MxD134 dataset. The *x*-axis and *y*-axis represent the 20 different amino acids and their relative proportions in the composition.

We would like to highlight that the amino acid residue compositions may vary in different datasets as well as within short (≤ 30 residues) and long (> 30 residues) disordered regions [28, 29]. Specifically, short disordered regions are enriched with aspartic acid (D), glycine (G) and serine (S). On the contrary, glutamic acid (E), lysine (K) and proline (P) are likely to be abundant in long disordered regions. To give further insight into this residue composition and confirm the ability of DisPredict to detect the residue preferences of short and long disordered regions, we determined the residual composition profile for our two test datasets, SL171 (Fig 10(A)) and MxD134 (Fig 10(B)). It is to be noted that, these two datasets contain experimentally annotated disorder from two different sources. SL171 contains sequences with disorder annotation from DisProt while MxD134 contains that from PDB. The composition profile consists of the actual ratio (*r*_*a*_) and predicted ratio (*r*_*p*_) of each amino acid type out of total annotated and predicted disordered residues.

The composition profile demonstrates that SL171’s disordered residue set accommodates relatively higher ratio of amino acid type E (10%) and K (9%), which are long disorder prone residues. In contrast, MxD134’s disordered residue set is enriched with high ratio of amino acid type S (11%), G (10%) and D (9%), known as short disorder prone residues. Another significant difference between the intrinsic compositions of these two datasets is in the proportion of histidine (H). Disorder annotation from PDB includes higher ratio of H-tag (8% in MxD134, compared to 2% in SL171), which is sometimes used for protein purification. The predicted proportion of all these amino acids given by DisPredict ensures its capability of detecting residues in disordered region of all length accurately with no significant over prediction. Moreover, DisPredict could also accurately predict methionine (M) at highly flexible N-terminal region. To further quantify DisPredict’s performance in detecting residue composition, we evaluated the Root Mean Square Difference (RMSE) and Pearson Correlation Coefficient (PCC) between actual and predicted ratio (*r*_*a*_ and *r*_*p*_) for each amino acid type. For MxD134 test dataset, we found RMSE of 0.0046, which was comparatively higher than the RMSE value computed for SL171 which equals to 0.0018. However, the correspondence between actual composition and predicted composition by DisPredict measured with PCC (P-Value < 10^−5^) was found equally positive, 0.9976 and 0.9897 for SL171 and MxD134 dataset, respectively. It is important to note that, this consistent result is corresponding to the independent test where the dataset used to train DisPredict shared significantly low sequence identity (at most 10%) with test dataset, which once again implicates the strength of the classification methodology of DisPredict.

Finally, accurate prediction of disorder has useful implication in proteomic studies due to its direct involvement in the proper function of a protein. Successful detection of disordered region of a protein is considered to be the first step in drug design to combat critical diseases. We have built DisPredict using the canonical SVM classifier with RBF kernel and established it as a successful fine predictor of disorder by utilizing the benchmark datasets. In addition to that, our case studies ensure biologically relevant performances of DisPredict.

## References

[1] Wright PE, Dyson HJ. Intrinsically unstructured proteins: re-assessing the protein structure-function paradigm. Journal of Molecular Biology. 1999;293: 321–331. 10.1006/jmbi.1999.3110 10550212

[2] Uversky VN, Dunker AK. Understanding protein non-folding. Biochimica Et Biophysica Acta (BBA)—Proteins And Proteomics. 2010;1804: 1231–1264. 10.1016/j.bbapap.2010.01.017 20117254 PMC2882790

[3] Uversky VN, Gillespie JR, Fink AL. Why are “natively unfolded” proteins unstructured under physiologic conditions? Proteins. 2000;41: 415–427. 10.1002/1097-0134(20001115)41:3<415::AID-PROT130>3.3.CO;2-Z 11025552

[4] Uversky VN. Natively unfolded proteins: A point where biology waits for physics. Protein Science. 2002;11: 739–756. 10.1110/ps.4210102 11910019 PMC2373528

[5] Tompa P. Intrinsically unstructured proteins. TRENDS in Biochemical Sciences. 2002;10: 527–533. 10.1016/S0968-0004(02)02169-2 12368089

[6] Dunker AK, Obradovic Z. The protein trinity–linking function and disorder. Nat Biotechnol. 2001;19: 805–806. 10.1038/nbt0901-805 11533628

[7] Vucetic S, Brown CJ, Dunker AK, Obradovic Z. Flavors of protein disorder. Proteins: Structure, Function, Bioinformatics. 2003;52: 573–584. 10.1002/prot.10437 12910457

[8] Radivojac P, Iakoucheva LM, Oldfield CJ, Obradovic Z, Uversky VN, Dunker AK. Intrinsic Disorder and Functional Proteomics. Biophysical Journal. 2007;92: 1493–1456. 10.1529/biophysj.106.094045 PMC1796814 17158572

[9] Whitford PC. Disorder guides protein function. Proc Natl Acad Sci USA. 2013;110: 7114–7115. 10.1073/pnas.1305236110 23610426 PMC3645555

[10] Dyson HJ, Wright PE. Coupling of folding and binding for unstructured proteins. Current opinion in structural biology. 2002;12: 54–60. 10.1016/S0959-440X(02)00289-0 11839490

[11] Uversky VN, Oldfield CJ, Dunker AK. Showing your ID: intrinsic disorder as an ID for recognition, regulation, cell signaling. J. Mol. Recogn. 2005;18: 343–384. 10.1002/jmr.747 16094605

[12] Dunker AK, Brown CJ, Obradovic Z. Identification and functions of usefully disordered proteins. Adv. Protein Chem. 2002;62: 25–49. 10.1016/S0065-3233(02)62004-2 12418100

[13] Dunker AK, Brown CJ, Lawson JD, Iakoucheva LM, Obradovic Z. Intrinsic disorder and protein function. Biochemistry. 2002;41: 6573–6582. 10.1021/bi012159+ 12022860

[14] Xue B, Dunker AK, Uversky VN. The Roles of Intrinsic Disorder in Orchestrating the Wnt-Pathway. Journal of Biomolecular Structure and Dynamics. 2012;29: 843–861. 10.1080/073911012010525024 22292947

[15] Kulkarni P, Rajagopalan K, Yeater D, Getzenberg RH. Protein folding and the order/disorder paradox. J Cell Biochem. 2011;112: 1949–1952. 10.1002/jcb.23115 21445877 PMC3472416

[16] Uversky VN, Oldfield CJ, Midic U, Xie H, Xue B, Vucetic S, et al Unfoldomics of human diseases: linking protein intrinsic disorder with diseases. BMC Genomics. 2009;10: S1–S7. 10.1186/1471-2164-10-S1-S7 PMC2709268 19594884

[17] Babu MM, Lee R, Groot NS, Gsponer J. Intrinsically disordered proteins: regulation and disease. Current Opinion in Structural Biology. 2011;21: 432–440. 10.1016/j.sbi.2011.03.011 21514144

[18] Cheng Y, LeGall T, Oldfield CJ, Mueller JP, Van Y-YJ, Romero P, et al Rational drug design via intrinsically disordered protein. Trends Biotechnol. 2006;24: 435–442. 10.1016/j.tibtech.2006.07.005 16876893

[19] Berman HM, Westbrook J, Feng Z, Gilliland G, Bhat TN, Weissig H, et al The Protein Data Bank. Nucleic Acids Res. 1999;28: 235–242. 10.1093/nar/28.1.235 PMC102472 10592235

[20] Obradovic Z, Peng K, Vucetic S, Radivojac P, Brown CJ, Dunker AK. Predicting intrinsic disorder from amino acid sequence. Proteins. 2003;53: 566–572. 10.1002/prot.10532 14579347

[21] Xue B, Dunbrack RL, Williams RW, Dunker AK, Uversky VN. PONDR-FIT: A Meta-Predictor of Intrinsically Disordered Amino Acids. Biochim Biophys Acta. 2010;1804: 996–101. 10.1016/j.bbapap.2010.01.011 20100603 PMC2882806

[22] Sickmeier M, Hamilton JA, LeGall T, Vacic V, Cortese MS, Tantos A, et al DisProt: the Database of Disordered Proteins. Nucleic Acids Res. 2007;35: 786–793. 10.1093/nar/gkl893 PMC1751543 17145717

[23] Fukuchi S, Amemiya T, Sakamoto S, Nobe Y, Hosoda K, Kado Y, et al IDEAL in 2014 illustrates interaction networks composed of intrinsically disordered proteins and their binding partners. Nucleic Acids Res. 2014;42: D320–D325. 10.1093/nar/gkt1010 24178034 PMC3965115

[24] Fukuchi S, Sakamoto S, Nobe Y, Murakami SD, Amemiya T, Hosoda K, et al IDEAL: Intrinsically Disordered proteins with Extensive Annotations and Literature. Nucleic Acids Res. 2012;40: D507–D511. 10.1093/nar/gkr884 22067451 PMC3245138

[25] Potenza E, Domenico TD, Walsh I, Tosatto SCE. MobiDB 2.0: an improved database of intrinsically disordered and mobile proteins. Nucl. Acids Res. 2014;43: D315–D320. 10.1093/nar/gku982 25361972 PMC4384034

[26] Domenico TD, Walsh I, Martin AJM, Tosatto SCE. MobiDB: a comprehensive database of intrinsic protein disorder annotations. Bioinformatics. 2012;28(15): 2080–2081. 10.1093/bioinformatics/bts327 22661649

[27] Pruitt KD, Tatusova T, Maglott DR. NCBI Reference Sequence (RefSeq): a curated non-redundant sequence database of genomes, transcripts and proteins. Nucleic Acids Res. 2005;33: D501–D504. Available: ftp://ftp.ncbi.nlm.nih.gov/blast/db/. 10.1093/nar/gki025 15608248 PMC539979

[28] Radivojac P, Obradovic Z, Smith DK, Zhu G, Vucetic S, Brown CJ, et al Protein flexibility and intrinsic disorder. Protein Sci. 2004;10: 71–80. 10.1110/ps.03128904 PMC2286519 14691223

[29] Peng K, Vucetic S, Radivojac P, Brown CJ, Dunker AK, Obradovic Z. Optimizing long intrinsic disorder predictors with protein evolutionary information. J Bioinform Comput Biol. 2005;3: 35–60. 10.1142/S0219720005000886 15751111

[30] Peng K, Radivojac P, Vucetic S, Dunker AK, Obradovic Z. Length-dependent prediction of protein intrinsic disorder. BMC Bioinformatics. 2006;7: 208 10.1186/1471-2105-7-208 16618368 PMC1479845

[31] Jones DT, Ward JJ. Prediction of disordered regions in proteins from position specific score matrices. Proteins. 2003;53(Suppl 6): 573–578. 10.1002/prot.10528 14579348

[32] Ward JJ, McGuffin LJ, Bryson K, Buxton BF, Jones DT. The DISOPRED server for the prediction of protein disorder. Bioinformatics. 2004;20: 2138–2139. 10.1093/bioinformatics/bth195 15044227

[33] Linding R, Jensen LJ, Diella F, Bork P, Gibson TJ, Russell RB. Protein disorder prediction: implications for structural proteomics. Structure. 2003;11: 1453–1459. 10.1016/j.str.2003.10.002 14604535

[34] Cheng J, Sweredoski MJ, Baldi P. Accurate Prediction of Protein Disordered Regions by Mining Protein Structure Data. Data Mining and Knowledge Discovery. 2005;11: 213–222. 10.1007/s10618-005-0001-y

[35] Yang ZR, Thomson R, McNeil P, Esnouf RM. RONN: the bio-basis function neural network technique applied to the detection of natively disordered regions in proteins. Bioinformatics. 2005;21: 3369–3376. 10.1093/bioinformatics/bti534 15947016

[36] Vullo A, Bortolami O, Pollastri G, Tosatto SC. Spritz: a server for the prediction of intrinsically disordered regions in protein sequences using kernel machines. Nucleic Acids Res. 2006;34: W164–W168. 10.1093/nar/gkl166 16844983 PMC1538873

[37] Schlessinger A, Yachdav G, Rost B. PROFbval: predict flexible and rigid residues in proteins. Bioinformatics. 2006;22: 891–893. 10.1093/bioinformatics/btl032 16455751

[38] Su CT, Chen CY, Ou YY. Protein disorder prediction by condensed PSSM considering propensity for order or disorder. BMC Bioinformatics. 2006;7: 319–334. 10.1186/1471-2105-7-319 16796745 PMC1526762

[39] Su CT, Chen CY, Hsu CM. iPDA: integrated protein disorder analyzer. Nucleic Acids Res. 2007;35: W465–W472. 10.1093/nar/gkm353 17553839 PMC1933224

[40] Ishida T, Kinoshita K. PrDOS: prediction of disordered protein regions from amino acid sequence. Nucleic Acids Res. 2007;35: W460–W464. 10.1093/nar/gkm363 17567614 PMC1933209

[41] Shimizu K, Muraoka Y, Hirose S, Tomii K, Noguchi T. Predicting mostly disordered proteins by using structure-unknown protein data. BMC Bioinformatics. 2007;8: 78–92. 10.1186/1471-2105-8-78 17338828 PMC1838436

[42] Hirose S, Shimizu K, Kanai S, Kuroda Y, Noguchi T. POODLE-L: a two-level SVM prediction system for reliably predicting long disordered regions. Bioinformatics. 2007;23: 2046–2053. 10.1093/bioinformatics/btm302 17545177

[43] Schlessingera A, Liu J, Rost B. Natively Unstructured Loops Differ from Other Loops. Bioinformatics. 2007;3: e140–e151. 10.1371/journal.pcbi.0030140 PMC1924875 17658943

[44] Yang JY, Yang MQ. Predicting protein disorder by analyzing amino acid sequence. BMC Genomics. 2008;9: S8–S15. 10.1186/1471-2164-9-S2-S8 18831799 PMC2559898

[45] Wang L, Sauer UH. OnD-CRF: predicting order and disorder in proteins using [corrected] conditional random fields. Bioinformatics. 2008;24: 1401–1402. 10.1093/bioinformatics/btn132 18430742 PMC2387219

[46] Deng X, Eickholt J, Cheng J. PreDisorder: ab initio sequence-based prediction of protein disordered regions. BMC Bioinformatics. 2009;10: 436–441. 10.1186/1471-2105-10-436 20025768 PMC3087350

[47] Zhang T, Faraggi E, Xue B, Dunker AK, Uversky VN, Zhou Y. SPINE-D: accurate prediction of short and long disordered regions by a single neural-network based method. J Biomol Struct Dyn. 2012;29: 799–813. 10.1080/073911012010525022 22208280 PMC3297974

[48] Walsh I, Martin AJM, Domenico TD, Tosatto SCE. ESpritz: accurate and fast prediction of protein disorder. Bioinformatics. 2012;28: 503–509. 10.1093/bioinformatics/btr682 22190692

[49] Linding R, Russell RB, Neduva V, Gibson TJ. GlobPlot: Exploring protein sequences for globularity and disorder. Nucleic Acids Res. 2003;31: 3701–3708. 10.1093/nar/gkg519 12824398 PMC169197

[50] Dosztányi Z, Csizmok V, Tompa P, Simon I. IUPred: web server for the prediction of intrinsically unstructured regions of proteins based on estimated energy content. Bioinformatics. 2005;21: 3433–3434. 10.1093/bioinformatics/bti541 15955779

[51] Prilusky J, Felder CE, Zeev-Ben-Mordehai T, Rydberg EH, Man O, Beckmann JS, et al FoldIndex: a simple tool to predict whether a given protein sequence is intrinsically unfolded. Bioinformatics. 2005;21: 3435–3438. 10.1093/bioinformatics/bti537 15955783

[52] Schlessinger A, Punta M, Rost B. Natively unstructured regions in proteins identified from contact predictions. Bioinformatics. 2007;23: 2376–2384. 10.1093/bioinformatics/btm349 17709338

[53] McGuffin LJ. Intrinsic disorder prediction from the analysis of multiple protein fold recognition models. Bioinformatics. 2008;24: 1798–1804. 10.1093/bioinformatics/btn326 18579567

[54] Ishida T, Kinoshita K. Prediction of disordered regions in proteins based on the meta approach. Bioinformatics. 2008;24: 1344–1348. 10.1093/bioinformatics/btn195 18426805

[55] Schlessinger A, Punta M, Yachdav G, Kajan L, Rost B. Improved Disorder Prediction by Combination of Orthogonal Approaches. PLoS One. 2009;4: e4433–e4442. 10.1371/journal.pone.0004433 19209228 PMC2635965

[56] Mizianty MJ, Stach W, Chen K, Kedarisetti KD, Disfani FM, Kurgan L. Improved sequence-based prediction of disordered regions with multilayer fusion of multiple information sources. Bioinformatics. 2010;26: i489–i496. 10.1093/bioinformatics/btq373 20823312 PMC2935446

[57] Mizianty MJ, Peng Z, Kurgan L. MFDp2: Accurate predictor of disorder in proteins by fusion of disorder probabilities, content and profiles. Intrinsically Disordered Proteins. 2013;1: e24428 10.4161/idp.24428 28516009 PMC5424793

[58] Iqbal S, Hoque MT. DisPredict: A Fine Disorder-Protein Predictor. Tech. Report. 2014;1. Available: http://cs.uno.edu/~tamjid/TechReport/DisPredict.pdf.

[59] Sirota FL, Ooi HS, Gattermayer T, Schneider G, Eisenhaber F, Maurer-Stroh S. Parameterization of disorder predictors for large-scale applications requiring high specificity by using an extended benchmark dataset. BMC Genomics. 2009;11: S15 10.1186/1471-2164-11-S1-S15 PMC2822529 20158872

[60] Altschul SF, Gish W, Miller W, Myers EW, Lipman DJ. Basic local alignment search tool. J Mol Biol. 1990;215: 403–410. Available: ftp://ftp.ncbi.nlm.nih.gov/blast/executables/blast+/. 10.1016/S0022-2836(05)80360-2 2231712

[61] Peng K, Radivojac P, Vucetic S, Dunker AK, Obradovic Z. Length-dependent prediction of protein intrinsic disorder. BMC Bioinformatics. 2006;7: 208. 10.1186/1471-2105-7-208 PMC1479845 16618368

[62] Meiler J, Muller M, Zeidler A, Schmäschke F. Generation and evaluation of dimension-reduced amino acid parameter representations by artificial neural networks. J Mol Model. 2001;7: 360–369. 10.1007/s008940100038

[63] Su CT, Chen CY, Ou YY. Protein disorder prediction by condensed PSSM considering propensity for order or disorder. BMC Bioinformatics:;7: 319–334. 10.1186/1471-2105-7-319 16796745 PMC1526762

[64] Pruitt KD, Tatusova T, Klimke W, Maglott DR. NCBI Reference Sequences: current status, policy and new initiatives. Nucleic Acids Res. 2009;37: D32–D35. Available: ftp://ftp.ncbi.nlm.nih.gov/blast/db/. 10.1093/nar/gkn721 18927115 PMC2686572

[65] Faraggi E, Zhang T, Yang Y, Kurgan L, Zhou Y. SPINE X: improving protein secondary structure prediction by multistep learning coupled with prediction of solvent accessible surface area and backbone torsion angles. J Comput Chem. 2012;33: 259–267. 10.1002/jcc.21968 22045506 PMC3240697

[66] Faraggi E, Xue B, Zhou Y. Improving the prediction accuracy of residue solvent accessibility and real-value backbone torsion angles of proteins by guided-learning through a two-layer neural network. Proteins. 2009;74: 847–856. 10.1002/prot.22193 18704931 PMC2635924

[67] Zhang T, Faraggi E, Zhou Y. Fluctuations of backbone torsion angles obtained from NMR-determined structures and their prediction. Proteins. 2010;78: 3353–3362. 10.1002/prot.22842 20818661 PMC2976825

[68] Iqbal S, Mishra A, Hoque MT. Improved prediction of accessible surface area results in efficient energy function application. J Theor Biol. 2015;380: 380–91. 10.1016/j.jtbi.2015.06.012 26092374

[69] Sharma A, Lyons J, Dehzangi A, Paliwal KK. A feature extraction technique using bi-gram probabilities of position specific scoring matrix for protein fold recognition. J Theor Biol. 2013;320: 41–46. 10.1016/j.jtbi.2012.12.008 23246717

[70] Sharma A, Dehzangi A, Lyons J, Imoto S, Miyano S, Nakai K, et al Evaluation of sequence features from intrinsically disordered regions for the estimation of protein function, PloS one. 2014;9: e89890 10.1371/journal.pone.0089890 24587103 PMC3933697

[71] Sun Y, Ming D. Energetic Frustrations in Protein Folding at Residue Resolution: A Homologous Simulation Study of Im9 Proteins. PLoS ONE. 2014;9: e97982 10.1371/journal.pone.0097982 PMC3909201 24498176

[72] Vendruscolo M, Paci E, Dobson CM, Karplus M. Three key residues form a critical contact network in a protein folding transition state. Letters to Nature. 2000;409: 641–645. 10.1038/35054591 11214326

[73] Chang C-C, Lin C-J. LIBSVM: A library for support vector machines. ACM Transactions on Intelligent Systems and Technology. 2011;2: 1–27. 10.1145/1961189.1961199

[74] Noivirt-Brik O, Prilusky J, Sussman JL. LAssessment of disorder predictions in CASP8. Proteins. 2009;77: 210–216. 10.1002/prot.22586 19774619

[75] Monastyrskyy B, Fidelis K, Moult J, Tramontano A, Kryshtafovych A. Evaluation of disorder predictions in CASP9. Proteins. 2011;79: 107–118. 10.1002/prot.23161 21928402 PMC3212657

[76] Monastyrskyy B, Kryshtafovych A, Moult J, Tramontano A, Fidelis K. Assessment of protein disorder region predictions in CASP10. Proteins. 2014;82: 127–137. 10.1002/prot.24391 23946100 PMC4406047

[77] Lobanov MY, Furletova EI, Bogatyreva NS, Roytberg MA, Galzitskaya OV. Library of disordered patterns in 3D protein structures, PLoS Comput. Biol. 2010;6: e1000958. 10.1371/journal.pcbi.1000958 PMC2954861 20976197

[78] DeLong ER, DeLong DM, Clarke-Pearson DL. Comparing the Areas under Two or More Correlated Receiver Operating Characteristic Curves: A Nonparametric Approach. Biometrics. 1988;44: 837–845. 10.2307/2531595 3203132

[79] Robin X, Turck N, Hainard A, Tiberti N, Lisacek F, Sanchez J-C, et al pROC: an open-source package for R and S+ to analyze and compare ROC curves. BMC Bioinformatics. 2011;12: 77 10.1186/1471-2105-12-77 21414208 PMC3068975

[80] Slupsky CM, Lisa NG, Donaldson LW, Mackereth CD, Seidel JJ, Graves BJ, et al Structure of the Ets-1 pointed domain and mitogen-activated protein kinase phosphorylation site. Proc. Natl. Acad. Sci. USA. 1998;95: 12129–12134. 10.1073/pnas.95.21.12129 9770451 PMC22796

[81] Baens M, Peeters P, Guo C, Aerssens J, Marynen P. Genomic organization of TEL: the human ETS-variant gene 6. Genome Res. 1996;6: 404–413. 10.1101/gr.6.5.404 8743990

[82] Colicelli J. Human RAS Superfamily Proteins and Related GTPases. Sci. STKE. 2004;250: re13. 10.1126/stke.2502004re13 PMC2828947 15367757

[83] Piskacek S, Gregor M, Nemethova M, Grabner M, Kovarik P, Piskacek M. Nine-amino-acid transactivation domain: establishment and prediction utilities. Genomics. 2007;89: 756–768. 10.1016/j.ygeno.2007.02.003 17467953

[84] McCoy M, Stavridi ES, Waterman JL, Wieczorek AM, Opella SJ, Halazonetis TD. Hydrophobic side-chain size is a determinant of the three-dimensional structure of the p53 oligomerization domain. EMBO J. 1997;16: 6230–6236. 10.1093/emboj/16.20.6230 9321402 PMC1326307

